# Iso-Seq analysis of the *Taxus cuspidata* transcriptome reveals the complexity of Taxol biosynthesis

**DOI:** 10.1186/s12870-019-1809-8

**Published:** 2019-05-21

**Authors:** Xuejun Kuang, Sijie Sun, Jianhe Wei, Ying Li, Chao Sun

**Affiliations:** Institute of Medicinal Plant Development (IMPLAD), Chinese Academy of Medical Sciences, No. 151, Malianwa North Road, Haidian District, Beijing, 100193 China

**Keywords:** *Taxus cuspidata*, Transcriptome, Iso-Seq, Taxol biosynthesis, Alternative splicing

## Abstract

**Background:**

*Taxus cuspidata* is well known worldwide for its ability to produce Taxol, one of the top-selling natural anticancer drugs. However, current Taxol production cannot match the increasing needs of the market, and novel strategies should be considered to increase the supply of Taxol. Since the biosynthetic mechanism of Taxol remains largely unknown, elucidating this pathway in detail will be very helpful in exploring alternative methods for Taxol production.

**Results:**

Here, we sequenced *Taxus cuspidata* transcriptomes with next-generation sequencing (NGS) and third-generation sequencing (TGS) platforms. After correction with Illumina reads and removal of redundant reads, more than 180,000 nonredundant transcripts were generated from the raw Iso-Seq data*.* Using Cogent software and an alignment-based method, we identified a total of 139 cytochrome P450s (CYP450s), 31 BAHD acyltransferases (ACTs) and 1940 transcription factors (TFs). Based on phylogenetic and coexpression analysis, we identified 9 CYP450s and 7 BAHD ACTs as potential lead candidates for Taxol biosynthesis and 6 TFs that are possibly involved in the regulation of this process. Using coexpression analysis of genes known to be involved in Taxol biosynthesis, we elucidated the stem biosynthetic pathway. In addition, we analyzed the expression patterns of 12 characterized genes in the Taxol pathway and speculated that the isoprene precursors for Taxol biosynthesis were mainly synthesized via the MEP pathway. In addition, we found and confirmed that the alternative splicing patterns of some genes varied in different tissues, which may be an important tissue-specific method of posttranscriptional regulation.

**Conclusions:**

A strategy was developed to generate corrected full-length or nearly full-length transcripts without assembly to ensure sequence accuracy, thus greatly improving the reliability of coexpression and phylogenetic analysis and greatly facilitating gene cloning and characterization. This strategy was successfully utilized to elucidate the Taxol biosynthetic pathway, which will greatly contribute to the goals of improving the Taxol content in *Taxus* spp. using molecular breeding or plant management strategies and synthesizing Taxol in microorganisms using synthetic biological technology.

**Electronic supplementary material:**

The online version of this article (10.1186/s12870-019-1809-8) contains supplementary material, which is available to authorized users.

## Background

*Taxus cuspidata*, an evergreen woody plant from the Taxaceae family that is native to Northeast China, Korea, Japan and the extreme southeast of Russia [1], has been deemed an endangered Tertiary relict species [1, 2]. However, *T. cuspidata* is well known worldwide for its ability to produce the antitumor metabolite Taxol, a complex tetracyclic diterpenoid that is mainly produced by plants from the *Taxus* genus [3]. Since Taxol was approved for the treatment of refractory ovarian cancer by the U.S. Food and Drug administration (FDA) in 1992, it has been increasingly adopted and is currently approved for the treatment of breast, lung, nonsmall cell lung cancer (NSCLC), Kaposi’s sarcoma, etc. [4, 5]. The annual sales of Taxol and its related products exceeded $100 million in 2016 [6]. Furthermore, because Taxol is currently one of the top-selling natural antitumor drugs, more widespread applications of the drug have created a severe supply and demand problem [7]. At present, Taxol is acquired mainly by two routes: direct extraction from the bark or needles of *Taxus* species and artificial semisynthesis from the extracted intermediates baccatin III or 10-deacetylbaccatin III (DAB) [7, 8]. Unfortunately, these two commercial methods face many challenges, including the slow growth and extremely low Taxol content of *Taxus* spp. and the high costs of purifying Taxol and its intermediates [7]. Therefore, current Taxol production cannot match the increasing needs of the market, and novel strategies should be considered to increase the Taxol supply, including *Taxus* cell culture, metabolic engineering and synthetic biology methods.

**Fig. 1:**
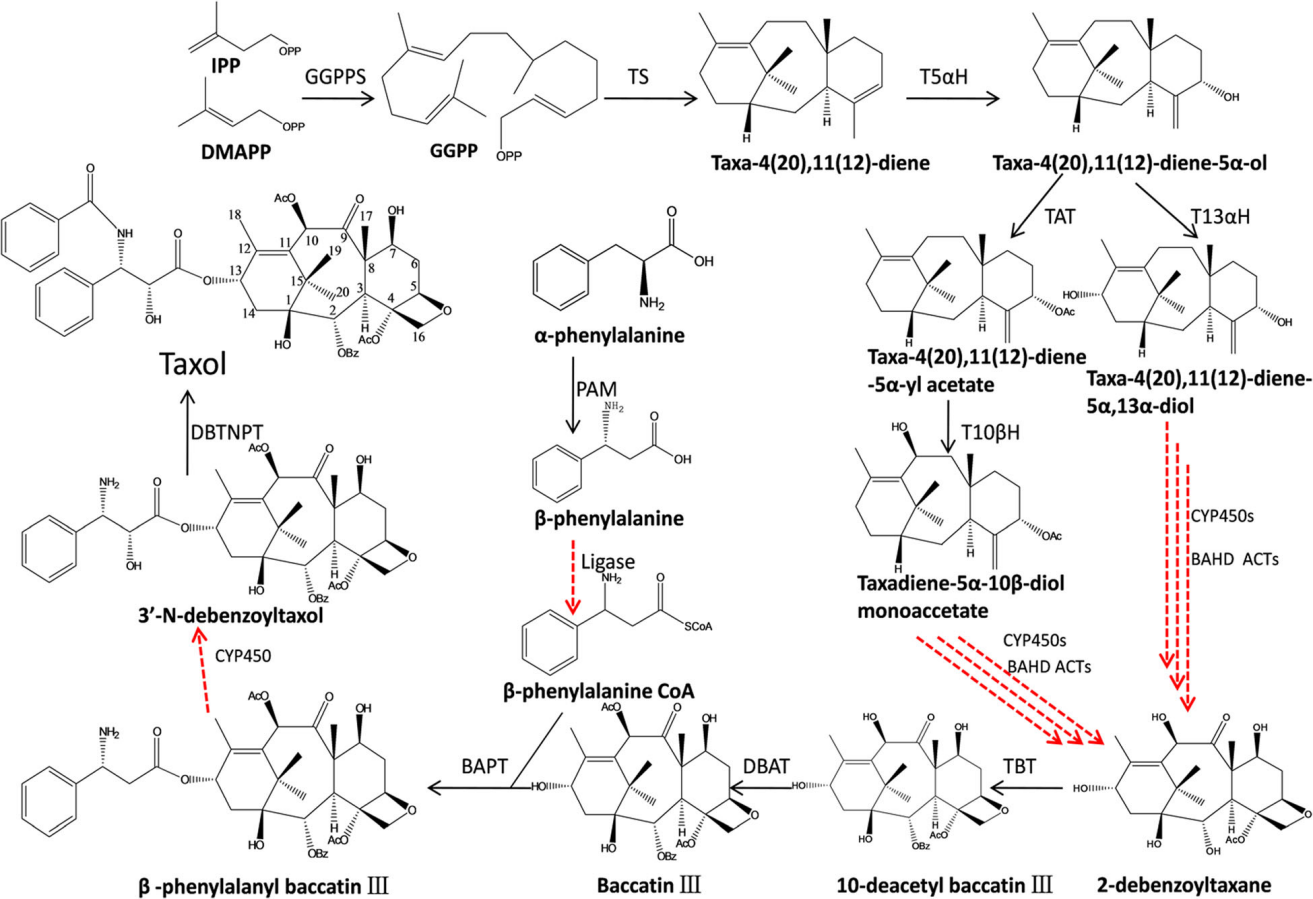
Putative Taxol biosynthetic pathway. The black solid arrows show the identified steps, and the red dotted arrows show the unknown steps. GGPPS: geranylgeranyl diphosphate synthase; TS: taxadiene synthase; T5αH: taxoid 5α–hydroxylase; TAT: taxadienol 5α-O-acetyl transferase; T13αH: taxoid 13α–hydroxylase; T10βH: taxoid 10β–hydroxylase; TBT: taxane-2α-O-benzoyltransferase; DBAT: 10-deacetylbaccatin III-10-O-acetyltransferase; PAM: phenylalanine aminomutase; BAPT: baccatin III-13-O-phenylpropanoyl transferase; DBTNBT: 30-N-debenzoyl-20-deoxytaxol-N-benzoyl transferase

Elucidating the biosynthetic pathway of Taxol in detail is essential in exploring alternative methods for Taxol production. In plants, all terpenoids arise from the common precursors dimethylallyl pyrophosphate (DMAPP) and isopentenyl diphosphate (IPP), which are typically synthesized by either the mevalonic acid (MVA) pathway in the cytoplasm or the methylerythritol phosphate (MEP) pathway in the plastid [9, 10]. For diterpenoid biosynthesis, one DMAPP unit and three IPP units can be condensed into geranylgeranyl diphosphate (GGPP) by geranylgeranyl diphosphate synthase (GGPPS) [9]. As shown in Fig. 1, the Taxol-specific synthetic branch starts from the cyclization of GGPP into the diterpene skeleton, termed taxadiene, and then multiple tailoring enzymes, mainly from the CYP450 and acyltransferase (ACT) families, are involved in modifying the skeleton [11, 12]. At least five CYP450s have been characterized in *Taxus* spp., which are responsible for hydroxylation at the C-2, C-5, C-7, C-10, and C-13 positions [13]. While the enzymes responsible for C-1 hydroxylation, C-9 oxidation, C-2′ hydroxylation and production of the oxetane ring are currently unknown, they are predicted to belong to the CYP450 family. Furthermore, five ACTs have been identified to be involved in Taxol biosynthesis, including taxadienol 5α-O-acetyl transferase (TAT), taxane-2α-O-benzoyltransferase (TBT), 10-deacetylbaccatin III-10-O-acetyltransferase (DBAT), BAPT (baccatin III-13-O-phenylpropanoyl transferase) and DBTNBT (30-N-debenzoyl-20-deoxytaxol-N-benzoyl transferase) [11]. All of these enzymes belong to a large group of plant-specific acyl-coenzyme A (acyl-CoA)-dependent ACTs, the so-called BAHD (acronym for the first four enzymes characterized: BEAT (benzylalcohol O-acetyltransferase), AHCT (anthocyanin O-hydroxycinnamoyltransferase), HCBT (anthranilate N-hydroxy-cinnamoyl/benzoyltransferase), and DAT (deacetylvindoline 4-O-acetyltransferase)) enzyme superfamily [11, 12]. However, several acetylation steps in the production of 2-debenzoyltaxane are still missing. Interestingly, the Taxol biosynthesis pathway may not be linear but rather a network of anastomosing routes that potentially have several common nodes. For example, both taxoid 2α-hydroxylase (T2αH) and taxoid 7β-hydroxylase (T7βH) can efficiently utilize taxusin as a substrate to form 2α-hydroxytaxusin and 7β-hydroxytaxusin, respectively [13]. Then, these intermediates can reciprocally convert the corresponding hydroxyl products of the respective reactions to the common 2α, 7β-dihydroxytaxusin. The complex biosynthetic network, unclear reaction orders and unobtainable taxane substrates have tremendously increased the difficulty of identifying the unknown enzymes in the pathway.

Understanding the regulatory mechanism of Taxol biosynthesis is a necessary prerequisite for improving the Taxol content in intact plants, tissues and cell cultures using biotechnology. Most transcription factors (TFs) are very important for regulating plant growth and development as well as for the biosynthesis of secondary metabolites [14, 15]. A number of TF families have been identified to participate in the regulation of Taxol biosynthesis, such as the WRKY, basic helix–loop–helix (bHLH), and AP2/ERF families [16–19]. For example, S. Li et al. [16] showed a WRKY TF, TcWRKY1, isolated from *T. chinensis* to be involved in transcriptional activation of the *DBAT* gene. Lenka et al. [17] demonstrated that three jasmonate-inducible bHLH TFs, TcJAMYC1, TcJAMYC2, and TcJAMYC4, from *T. cuspidata* play negative roles in Taxol biosynthesis. However, in some cases, TFs from the same families were shown to potentially play opposite roles in regulating Taxol biosynthesis. Zhang et al. [18] reported that two AP2/ERF TFs, TcERF12 and TcERF15, function as negative and positive regulators of the TS gene in *T. chinensis*. The functional diversity of TFs has led to difficulty in understanding the regulatory mechanism of Taxol biosynthesis.

Alternative splicing (AS) can produce multiple transcript isoforms from a single pre-mRNA via variable splice site selection [20]. In plants, the extent of intron-containing gene AS ranges from 42 to 61%, compared with 90–95% in humans [21]. Widespread changes in AS in response to environmental stress suggest its potential role in regulating the biosynthesis of secondary metabolites, which is regarded as a method of plant defense against biotic and abiotic stress [22]. Emerging evidence indicates that AS can not only regulate transcript levels by producing new, unstable mRNA isoforms that can be degraded by nonsense-mediated decay (NMD) but also produce alternate functional mRNAs encoding protein isoforms that differ in subcellular localization, stability, or function by changing or completely removing functional domains via the introduction of a premature termination codon (PTC), intron retention, or alternative 3′ or 5′ splice site selection [23]. However, little is known about the functions of AS in the regulation of Taxol biosynthesis due to the lack of AS information at the genomic level in *Taxus* spp.

Transcriptome analysis-based next-generation sequencing (NGS) technology is a powerful and economical way to obtain genetic information on a large scale and has been widely used to uncover genes involved in the biosynthesis of secondary metabolites [24, 25]. Although NGS has the advantages of high sequencing depth and low cost, the short read length generated may restrict correct sequence assembly and annotation [25]. Third-generation sequencing (TGS) technology, single-molecule real-time (SMRT) sequencing developed by the PacBio company, offers an alternative method for overcoming these limitations. Compared to NGS, SMRT sequencing yields longer reads and utilizes an assembly-free analysis pipeline, thus providing more full-length transcripts and direct evidence of the structural variation of isoforms [26]. Currently, long-read SMRT sequencing has been successfully applied to sequence the transcriptomes of several plant species, such as *Zea mays* [27], *Sorghum bicolor* [28], *Arabidopsis thaliana* [29], and strawberry [30].

Due to its large genome size, little information about the *T. cuspidata* genome is currently available, inhibiting elucidation of the Taxol biosynthetic pathway. Here, we developed a practical strategy to mine candidate genes involved in Taxol biosynthesis based on an Iso-Seq analysis of the *T. cuspidata* transcriptome. The candidate sequences and in-house pipelines produced from this study provide valuable resources for the elucidation of Taxol biosynthesis and will be beneficial for future studies on the production of Taxol or its precursors with synthetic biology technology.

## Methods

### Plant materials

*T. cuspidata* grown in Institute of Medicinal Plant Development served as the source of plant material in this study. Roots, stems and leaves were collected in three duplicates. After collection, all samples were immediately frozen in liquid nitrogen and stored at − 80 °C prior to RNA extraction. Total RNA was extracted using an RNAprep Plant Kit (Qiagen, Valencia, CA, USA) and quantified by Qubit (Invitrogen Life Technologies, USA). The RNA integrity was evaluated on an Agilent 2100 Bioanalyzer (Agilent Technologies, USA).

### Library preparation and Iso-Seq

Total RNA from different tissues was mixed at equal ratios. Poly(A) RNA was isolated from total RNA using Dynal oligo(dT)25 beads (Life Technologies, USA) and used for construction of the Iso-Seq library. The first cDNA strand was synthesized from purified polyA RNAs using the Clontech SMARTer PCR cDNA Synthesis Kit (Clontech, Mountain View, CA, USA). After PCR optimization, large-scale PCR was performed to synthesize the second cDNA strand without size selection. Equimolar mixed libraries of unfiltered fragments and > 4 kb fragments were prepared with the SMRTbell Template Prep Kit 1.0. Sequencing was performed on a PacBio Sequel platform. A total of four SMRT cells were utilized in this study.

### Iso-Seq data analysis

The raw data were processed using SMRTlink 4.0 software. Circular consistency sequences (CCSs) were generated from subread sequences by mutual correction and then classified into full-length or non-full-length reads by examining whether the 5′ primer, 3′ primer, or polyA tail was present. Full-length reads were corrected by isoform-level clustering (ICE) to obtain clustered consensus sequences, and then final arrow polishing was performed with non-full-length reads to obtain polished consensus sequences. Finally, the high-quality consensus transcripts of multiple libraries were merged, and redundant reads were removed based on CD-HIT-EST (−c 0.99) to obtain nonredundant transcripts [31]. The Coding Genome Reconstruction Tool (Cogent) was then used to further partition these error-corrected nonredundant transcripts into transcript families based on the k-mer clustering method [32]. Then, each transcript family was further reconstructed into one or several unique transcript models (UniTransModels) using the De Bruijn graph method. Benchmarking Universal Single-Copy Orthologs (BUSCO) [33] was used to evaluate the integrity of the transcriptome without redundancy, and the number of Embryophyta gene sets used in this evaluation was 1440.

### RNA-Seq and data analysis

A total of twelve RNA samples from the roots, stems and leaves were used in four duplicates to construct the transcriptome sequencing library. The transcriptome library was pair-end sequenced on the Illumina HiSeq™ 2000 platform. Clean reads were used for error correction to obtain the polished consensus sequences as described above. For comparison of Iso-Seq and RNA-Seq data, Illumina data from the same samples were assembled with Trinity and SOAP to produce unigenes. Coding sequences (CDSs) from RNA-Seq unigenes predicted using Swiss-Prot and NCBI Non-redundant Protein (Nr) data and the CDSs from unigenes assembled by Cogent using ANGEL software [34] were compared.

### Gene functional annotation and differential expression analysis

Gene functions were annotated using the following databases: Kyoto Encyclopedia of Genes and Genomes (KEGG) (http://www.kegg.jp/) [35], Swiss-Prot (https://www.uniprot.org/uniprot/) [36], Pfam (https://pfam.xfam.org) [37], euKaryotic Ortholog Groups (KOG) (ftp://ftp.ncbi.nih.gov/pub/COG/KOG/) [38], NCBI Non-redundant Protein (Nr) (https://www.ncbi.nlm.nih.gov/protein/) [39], NCBI Non-redundant Nucleotide (Nt)(https://www.ncbi.nlm.nih.gov/nucleotide/) and Gene Ontology (GO)(http://www.geneontology.org/) [40]. The expression analysis of unigenes in different tissues was carried out using RSEM (v1.1.12) software [41] with Illumina reads. The heatmap was plotted using pheatmap version 1.0.8. (https://CRAN.R-project.org/package=pheatmap).

### Noncoding RNA analysis

Several tools have been used to evaluate the coding potential of unigenes, such as Coding Potential Calculator (CPC) [42], Coding-Non-Coding Index (CNCI) [43] and Pfam protein structure domain analysis [37]. CNCI profiles adjoin nucleotide triplets to effectively distinguish protein-coding and noncoding sequences independent of known annotations. CPC mainly assesses the extent and quality of the open reading frame (ORF) in a transcript and searches the sequences with NCBI eukaryote protein databases to clarify the coding and noncoding transcripts, and an e-value of ‘1e-10’ was used in our analysis. We translated each transcript into all three possible frames and used Pfam Scan to identify the occurrence of any of the known protein family domains documented in the Pfam database. Any transcript with a Pfam hit was excluded from the following steps. Pfam searches use the default parameters -E 0.001 --domE 0.001.

### Identification of unigenes related to the Taxol pathway

Based on functional annotations from the Swiss-Prot and Pfam databases, the unigenes of CYP450s were identified in *T. cuspidata*. The classification of TcuCYP450 proteins was based on reference sequences from a P450 database established by Nelson. BAHD ACTs were identified by searching for the key word “PF02458” in the Pfam database. Unigenes were submitted to the iTAK online program (version 1.7.0b) [44] for identification and classification of TFs. The genome databases of *Arabidopsis thaliana* and *Salvia miltiorrhiza* used for TF comparative analysis were derived from NCBI.

### Phylogenetic and structural analyses

The CYP450, WRKY, bHLH and ERF phylogenetic trees were constructed using the neighbor-joining (NJ) method with the “Poisson correction” and “pairwise deletion of gaps” functions in MEGA6 software [45]. The significance level for the phylogenetic tree was assessed by bootstrap testing with 1000 replications. The BAHD ACT phylogenetic trees were constructed using the maximum likelihood (ML) method. The accession numbers of protein sequences derived from GenBank are listed in Additional file 1: Table S1.

### Real-time PCR

RNA samples were isolated from the roots, stems and leaves in three biological replicates. Reverse transcription was performed using the GoScript™ Reverse Transcription System kit (Promega, USA). For each sample, reverse transcription was performed using 2 μg of total RNA and 200 U M-MLV Transcriptase (Promega) in a 40 μl volume. The reaction was carried out at 25 °C for 5 min, 42 °C for 60 min and 70 °C for 15 min. A qPCR analysis was then conducted in triplicate using SYBR Premix Ex Taq (Takara®, Tokyo, Japan) and a 7500 Real-time PCR system (ABI). The reaction mixture (20 μL) contained 10 μL of 2 × SYBR Premix Ex Taq, 0.5 μL of each forward and reverse primer, and 1 μL of template cDNA. PCR amplification was performed under the following conditions: 95 °C for 30 s; 40 cycles of 95 °C for 5 s, 60 °C for 30 s and 72 °C for 15 s; and 95 °C for 10 s. The primers used in this study are listed in Additional file 2: Table S2. The relative expression levels were calculated using the 2^–△△Ct^ method.

## Results

### Transcriptome sequencing and annotation

**Fig. 2:**
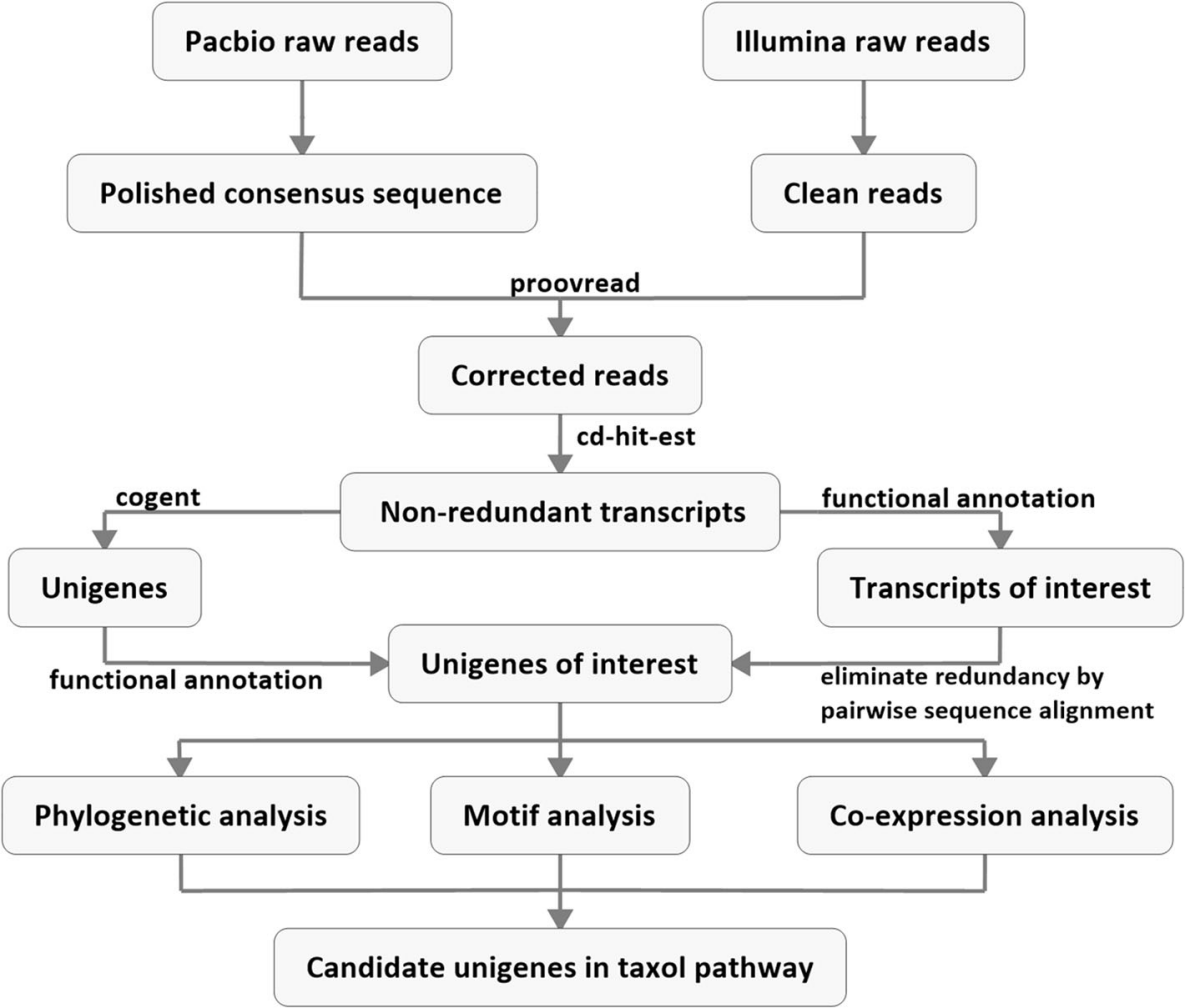
Strategy for mining candidate genes involved in the Taxol biosynthesis pathway

To identify as many transcripts as possible, equal amounts of total RNA from the roots, stems and leaves of *T. cuspidata* plants were pooled together and reverse transcribed into cDNA. To minimize bias that favors sequencing shorter transcripts, unfiltered and > 4 kb cDNA fragments were equally mixed and used to construct sequencing libraries. Using the PacBio Sequel platform, a total of 5,678,524 subreads with an average length of 2047 bp were generated. According to the bioinformatics procedure shown in Figs. 2, 568,432 CCSs were obtained from the subreads by removing adapters and artifacts. After read clustering and consensus calling, 181,230 sequences were retained as final consensus transcripts for subsequent analysis, which included evidenced-based gene model construction and candidate gene discovery. These consensus transcripts were further corrected with Illumina short reads to improve the accuracy (Proovread), and redundant transcripts were then removed with CD-HIT software, generating 148,038 nonredundant transcripts. Finally, Cogent software was used to reconstruct gene models and visualize the AS. Among all nonredundant transcripts, 103,072 transcripts were reconstructed into 13,636 UniTransModels, and 44,966 transcripts were left as singletons, which had only one splicing type. In total, 58,602 unigenes (UniTransModels + singletons) were obtained, each of which was bioinformatically predicted to be one genomic locus. The completeness of the transcriptome was assessed by the BUSCO method with the Embryophyta (ODB10) core gene dataset. Approximately 91.1% of the 1440 expected embryophyte genes were identified as complete, indicating the high integrity of the *T. cuspidata* transcriptome.

**Fig. 3:**
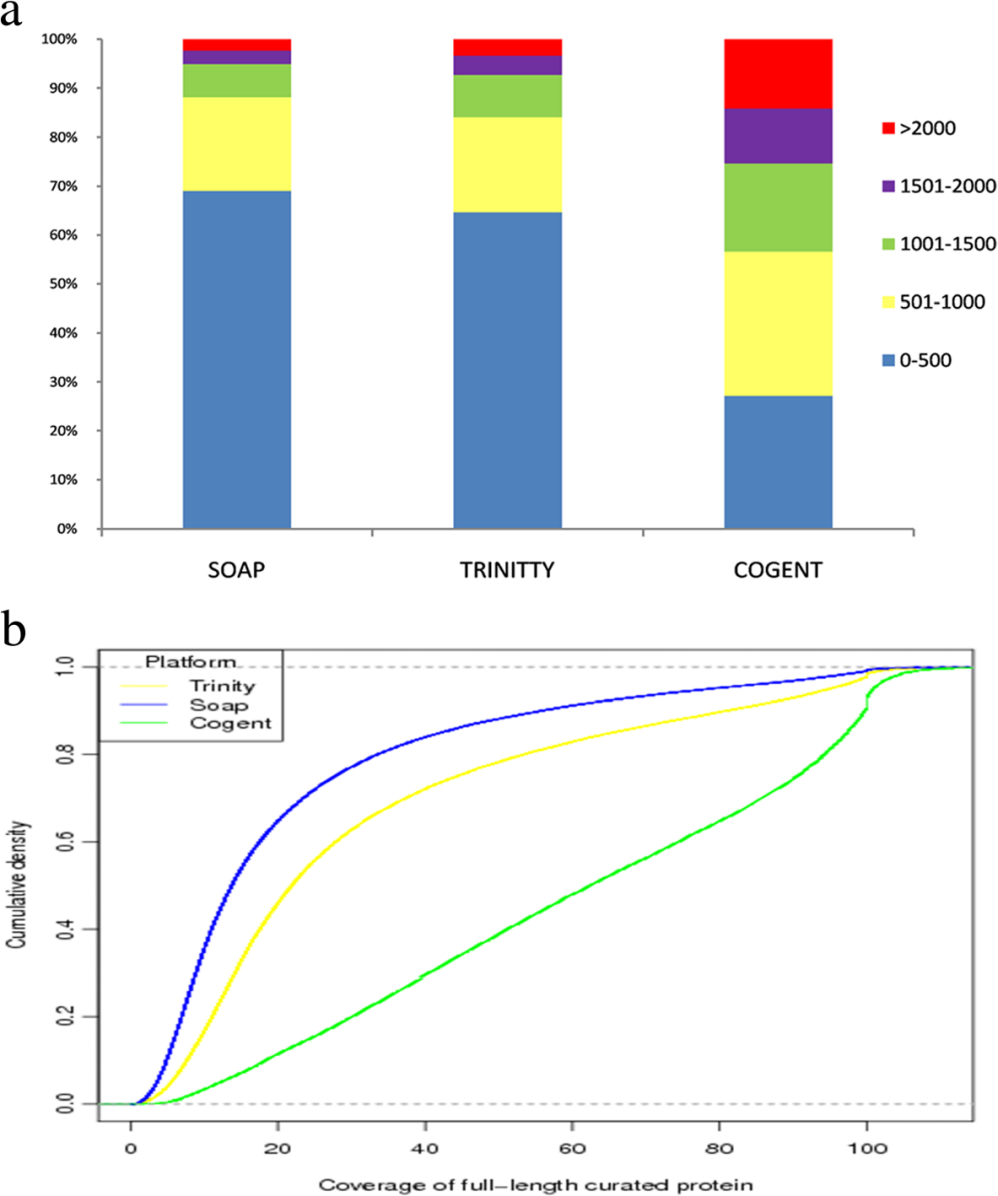
Comparison of *T. cuspidata* transcriptomes acquired using different sequencing platforms. **a** Length distribution of unigenes from the SOAP, Trinity and Iso-Seq pipelines. **b** Cumulative density plot showing the coverage of full-length curated proteins (Swiss-Prot) for unigenes produced by different sequencing platforms

To compare the integrality of transcripts from Iso-Seq and RNA-Seq, RNA-Seq data from the same samples were assembled with Trinity and SOAP, producing 63,872 and 53,781 unigenes, respectively. As shown in Fig. 3a, the Iso-Seq unigenes had much longer CDSs than those from RNA-Seq. For unigenes produced by Trinity and SOAP, CDSs less than 1000 bp accounted for 84.1 and 88.1% of the total, respectively, numbers obviously higher than those generated by Cogent software (56.5%). For unigenes from Cogent, the CDSs with more than 2000 bp accounted for 14.2% of the total, compared with Trinity (3.4%) and SOAP (2.3%). Next, we further investigated the transcript integrality by aligning the unigenes from the different sequencing platforms to those of a well-curated full-length protein database, UniProt Swiss-Prot (UniProtKB). Compared to the RNA-Seq unigenes, a significantly increased percentage of the Iso-Seq unigenes contained full-length ORFs (covering 100% of curated full-length proteins) or nearly full-length ORFs (covering 90% of curated full-length proteins) (Fig. 3b). We compared our 58,602 unigenes with 18 genes encoding enzymes determined to be involved in Taxol biosynthesis and found that most (14 out of 18) were nearly fully represented by long-read sequences (95% coverage). These results suggested that Iso-Seq confers a substantial advantage in the production of full-length transcripts over RNA-Seq.

To capture the most informative and complete annotation information, we used a basic local alignment search tool (BLAST) to annotate all the unigenes based on sequence similarity searches against public databases, including the Swiss-Prot, KEGG, GO, KOG, NCBI Nr, and NCBI Nt databases. In addition, annotation was performed with hmmScan based on a domain similarity search against the Pfam database. In total, 42,920 unigenes were successfully matched to known sequences or domains in at least one of the seven databases, and 10,388 unigenes were annotated in all the databases (Additional file 4: Figure S1). In addition, a total of 9523 transcripts were predicted to be high-confidence lncRNAs by CPC, CNCI and Pfam protein structure domain analysis (Additional file 5: Figure S2). LncRNAs are important regulators of the secondary metabolism pathway, regulating gene expression on multiple levels via a number of complex mechanisms [46, 47].

To functionally classify the *T. cuspidata* unigenes, GO terms were assigned to each unigene using BLAST2GO based on the best BLASTx hit from the NR database. In total, 23,966 unigenes were assigned GO terms, which were classified into three major categories (biological process, cellular component and molecular function) (Additional file 6: Figure S3). The major subgroups of biological processes were “cellular process” (GO: 0009987) and “metabolic process” (GO: 0008152). In the cellular component category, unigenes involved in the “cell part” (5120, 22.2% of the total) and “cell” (5120, 22.2%) were highly represented. For the molecular function classification, the major categories were “binding” (GO: 0005488) and “catalytic activity” (GO: 0003824). Additionally, to explore the biological functions and interactions of unigenes in *T. cuspidata*, 58,602 unigenes were searched against the KEGG database. A total of 38,547 unigenes had significant matches in the database and were assigned to 363 KEGG pathways, which were categorized into five subcategories as follows: organismal systems, genetic information processing, cellular process, environmental information processing, and metabolism (Additional file 7: Figure S4). The most heavily enriched KEGG pathways were related to metabolic pathways. Among these pathways, the “biosynthesis of secondary metabolites” pathway included 8155 unigenes, providing a valuable resource for further gene function research. The pathways with the highest unigene representations were those related to carbon metabolism (ko01200; 496 unigenes), biosynthesis of amino acids (ko01230; 449 unigenes), purine metabolism (ko00230; 283 unigenes), oxidative phosphorylation (ko00190; 235 unigenes), and amino sugar and nucleotide sugar metabolism (ko00520, 224 unigenes).

### Gene expression analysis

**Fig. 4:**
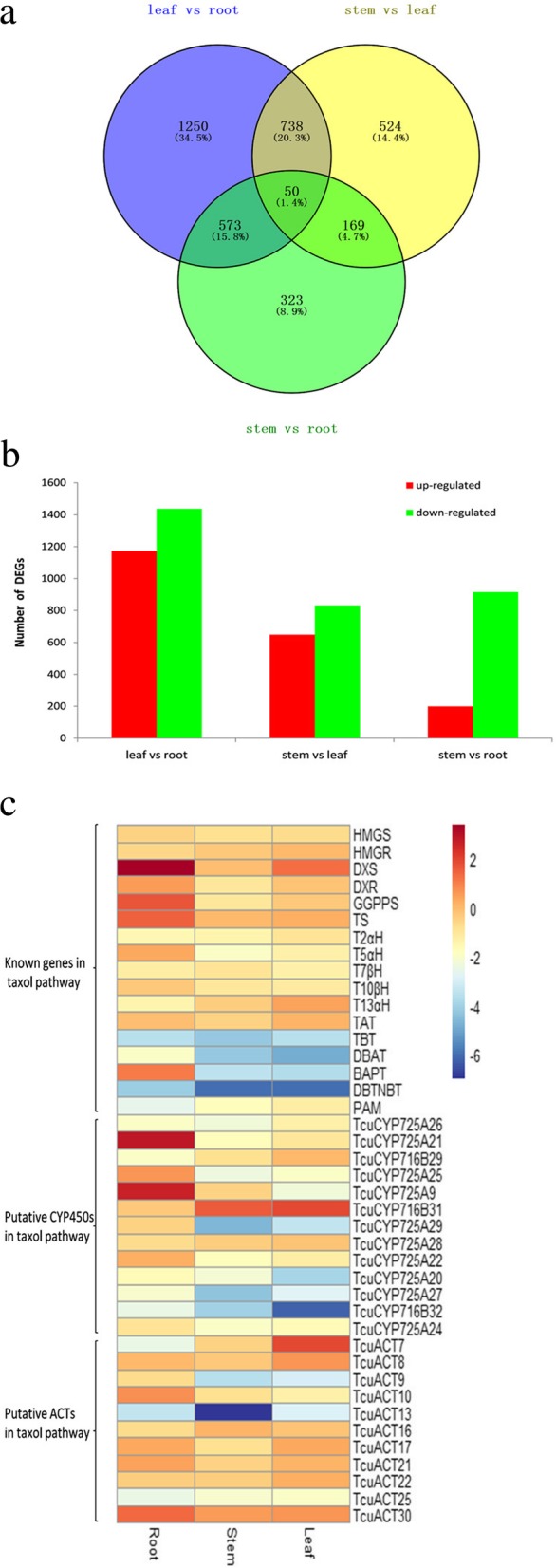
The expression patterns of unigenes **a** Venn diagram of differentially expressed unigenes. **b** Changes in gene expression profiles among the different *Taxus* tissues. **c** Expression analysis of identified genes and candidate unigenes in the Taxol pathway by real-time PCR. Heatmap of expression levels based on their real-time PCR values in three tissues, including roots, stems, and leaves

To identify gene expression differences in different *Taxus* tissues, we analyzed the expression patterns of 58,602 unigenes and found 3627 differentially expressed genes (DEGs) (Fig. 4a). The largest differences were observed between leaves and roots, in which 2611 DEGs were detected, including 1437 upregulated unigenes and 1174 downregulated unigenes. The smallest difference existed between stems and roots, in which 1115 DEGs were detected, including 199 upregulated unigenes and 916 downregulated unigenes. Between stems and leaves, 1482 DEGs were detected, including 649 upregulated unigenes and 832 downregulated unigenes, and 50 unigenes were differentially expressed in any two of the three tissues (Fig. 4b). In other words, the expression differences were more remarkable in the leaf versus root comparison than in the leaf versus stem and stem versus root comparisons. The functional categories of 402 total unigenes upregulated in roots were further analyzed using the KEGG database, and the significant DEGs were represented in the five main KEGG categories. Among them, 227 unigenes were associated with secondary metabolism (Additional file 8: Figure S5). The biosynthesis of secondary metabolites and plant hormone signal transduction were each significantly enriched in the unigenes specifically expressed in roots. In addition, 161 DEGs belong to TFs, 104 of which were upregulated in roots.

As shown in Fig. 1, the biosynthesis of Taxol is a complex process because skeleton modifications require the participation of several hydroxylases, ACTs and mutases. To investigate whether the genes involved in Taxol biosynthesis are coexpressed, we analyzed the expression patterns of 12 characterized genes in this pathway using real-time PCR. As shown in Fig. 4c and Additional file 3: Table S3 qRT-PCR results are, in most cases, consistent with the expression levels calculated from RNA-Seq data. The gene encoding the signature enzyme TS was obviously expressed at a higher level in roots than in stems and leaves, while its expression in leaves was slightly higher than that in stems (Fig. 4c). The *PAM* gene, involved in side chain synthesis, exhibited expression patterns different from those of *TS*, as *PAM* was expressed at the lowest level in the root, followed by the stem, and exhibited the highest expression in the leaf. Among ten genes encoding enzymes modifying the Taxol skeleton, six had expression patterns similar to those of *TS*. In the left four genes, *TAT* showed high expression in all three tissues, while *T2αH* and *T7βH* showed moderate expression in all tissues; *T13αH* exhibited an expression trend opposite that of *TS*, showing the lowest expression in the root and the highest expression in the leaf. According to our coexpression analysis, we hypothesized that the enzymes involved in the stem biosynthetic pathway are TS, T5αH, TAT, T10βH, TBT, DBAT, BAPT and DBTNPT. In addition, several genes in the first part of the biosynthetic pathway were expressed at substantially higher levels than those in the final steps. In particular, the gene encoding the last enzyme, DBTNPT, was expressed at the lowest level in all plant tissues, which is one of the reasons underlying the extremely low Taxol content in *T. cuspidata*. Therefore, we hypothesized that dramatically improving *DBTNPT* expression will substantially contribute to Taxol production. We also investigated the coexpression of Taxol-specific genes with several upstream genes and found that *GGPPS* and two genes from the MEP pathway, *DXS* and *DXR*, were consistently coexpressed with most of the downstream genes, such as *TS*, *T5αH*, *T10βH*, *TBT*, *DBAT*, *BAPT* and *DBTNPT*, while *HMGS* and *HMGR* from the MVA pathway had expression patterns different from those downstream genes, suggesting that the isoprene precursors for Taxol biosynthesis may primarily derive from the MEP pathway, which is consistent with previous reports [48].

### Alternative splicing analysis

**Fig. 5:**
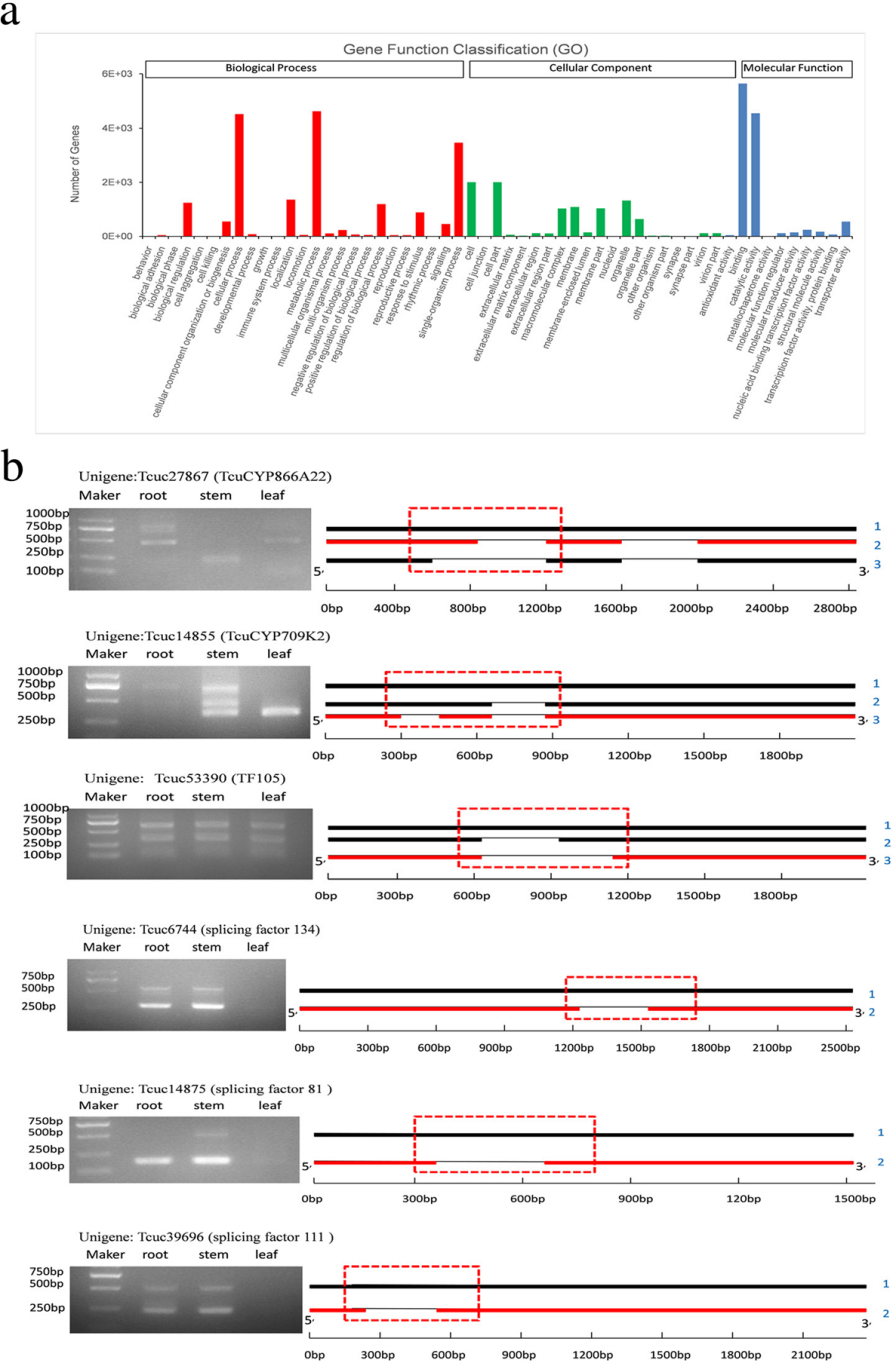
Sketch maps of alternative splicing forms. **a** GO annotation of unigenes containing AS. **b** AS was validated via RT-PCR. The red dotted boxes show the fragments amplified with a specific primer. The red bold lines show the isoforms with the correct ORFs in each gene. The blue numbers on the right show the different transcripts of unigenes

AS is a regulated process that increases the diversity of an organism’s transcriptome and proteome, mediating plant biological processes ranging from plant development to stress responses [21, 22]. In our results, transcript isoforms were identified by aligning individual long-read consensus transcripts back to the reconstructed full-length unigenes. We identified 13,636 unigenes undergoing AS events, of which 8627 were assigned GO terms, which were classified into three major categories (biological process, cellular component and molecular function) (Fig. 5a). GO enrichment analysis showed that these AS genes are highly enriched in binding, catalytic activity, metabolic processes and cellular processes.

Six unigenes were used to validate the authenticity of the AS events using the RT-PCR method, including two genes encoding CYP450s, one encoding a TF and three encoding splicing factors (Fig. 5b). Specific primers were designed to amplify the fragments of the predicted transcripts. The PCR fragments matching the predicted sizes were subsequently sequenced with the Sanger sequencing method. All the AS events were confirmed, suggesting the reliability of our analytical procedure even in the condition without an available reference genome. Surprisingly, the AS of some genes exhibits a tissue-preferential pattern. For example, only isoform 3 of TcuCYP709K2 (Tcuc14855), which has a correct ORF and can be translated into a functional protein, was found in the leaf, while three isoforms existed in the stem. For unigene TcuCYP866A22 (Tcuc27867), the leaves produced mainly isoform 2, while the stem produced isoform 3, and the root produced isoform 1 and isoform 2. Isoform 2 had a correct ORF and the ability to synthesize active protein. The molecular mechanism underlying the tissue-preferential pattern of AS and its role in regulating gene expression and encoded protein function require further study.

### Putative CYP450 genes involved in Taxol biosynthesis

Plant CYP450s are heme-containing enzymes that play roles in a wide variety of both primary and secondary metabolism reactions [49–51]. To date, five CYP450s have been shown to be involved in Taxol biosynthesis, and at least three missing steps in the pathway are thought to be catalyzed by enzymes from the CYP450 superfamily. Based on the annotation results, a total of 139 full-length or near full-length CYP450s (458 ~ 673 amino acids in length) with intact Pfam CYP450 domains were classified by alignment with the CYP450 database using standard sequence similarity cutoffs, specifically 40, 55 and 97% for family, subfamily and allelic variants, respectively. Thus, the 139 TcuCYP450s were classified into 36 families and 63 subfamilies (Additional file 9: Figure S6). Among 139 members, 79 TcuCYP450s were identified for the first time in *T. cuspidata,* and 66 TcuCYP450s were gymnosperm-specific CYP450s from 6 families (CYP750, CYP867, CYP725, CYP947, CYP864 and CYP866) and 3 subfamilies (CYP76AA, CYP720B and CYP716B).

**Fig. 6:**
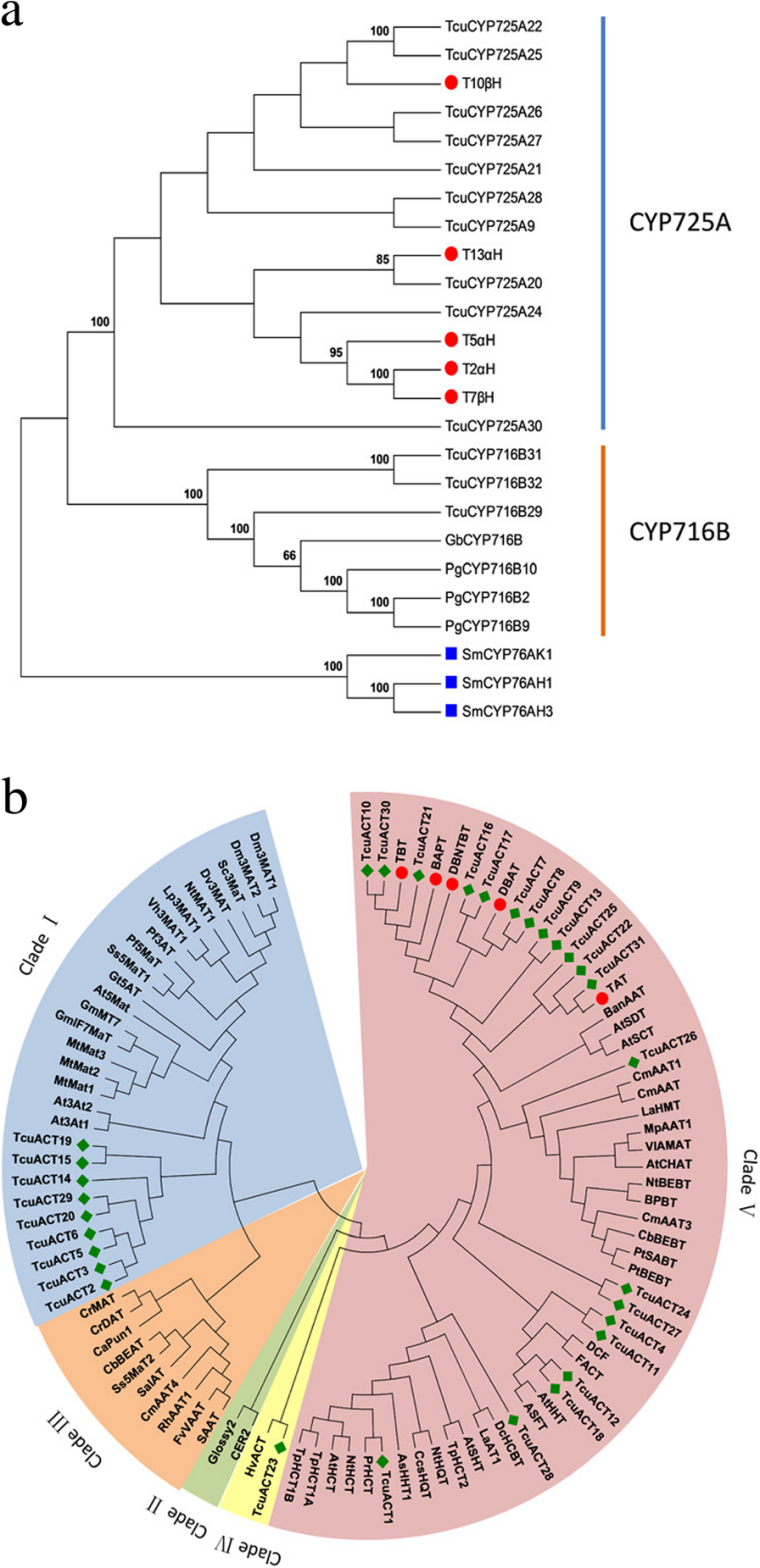
Phylogenetic analyses of CYP450s and BAHD ACTs. **a** Phylogenetic analyses of CYP725 and CYP716 family CYP450 proteins in *T. cuspidata* and *P. glauca*. **b** Phylogenetic analyses of BAHD ACTs in *T. cuspidata* and characterized BAHD ACTs from other plants. The filled green diamonds represent unigenes in *T. cuspidata*, the filled red circles indicate the identified genes in *Taxus* spp., and the filled blue square indicates the outgroup

Four previously characterized CYP450 genes encoding T2αH [52], taxadiene 5α-hydroxylase [53], taxoid 10β-hydroxylase [54] and taxoid 14β-hydroxylase [55] were found in our transcriptomes. To date, all of the characterized CYP450s involved in Taxol biosynthesis belong to the CYP725A subfamily. We inferred that 10 novel CYP725A unigenes may be candidate CYP450s of the Taxol pathway (Fig. 6a). Moreover, Zhang et al. [56] identified two candidate genes with high similarity to *Taxus* CYP450s via analysis of high-throughput RNA sequencing data from *G. biloba* and found that *G. biloba* suspension cells exhibit taxoid 9α-hydroxylation activity. This CYP450 belongs to the CYP716B subfamily, suggesting that C9 hydroxylases in *T. cuspidata* may belong to the CYP716B subfamily (Fig. 6a). Therefore, we chose to further analyze the expression patterns of 13 TcuCYP450 unigenes in different tissues, including 10 unigenes encoding enzymes from the CYP725A subfamily and 3 TcuCYP450 unigenes from the CYP716B subfamily (Fig. 4c). The qRT-PCR profiles showed that 8 novel unigenes in the CYP725A subfamily and one unigene in the CYP716B subfamily had tissue-specific expression patterns similar to those of known genes in the Taxol biosynthetic pathway (Fig. 4c). In particular, the expression levels of *TcuCYP725A21* and *TcuCYP725A9* in roots were more than 47-fold and 19-fold higher than those in leaves and 96-fold and 60-fold higher than those in stems, respectively. Considering all the evidence, most genes in the Taxol pathway were highly expressed in the root, and we inferred that the above 9 unigenes are candidates for Taxol biosynthesis.

### Putative BAHD ACTs in Taxol biosynthesis

Until now, five ACTs have been shown to participate in the Taxol biosynthetic pathway, including *TAT*, *TBT*, *DNTBAT*, *DBAT* and *BAPT*, and all belong to BAHD ACTs [11, 57–60]. These ACTs deliver an acyl group from a corresponding acyl-CoA thioester to a Taxol pathway intermediate, catalyzing either O- or N-acyl group transfer reactions. Some acetylases in the Taxol pathway remain unidentified, such as the enzyme that can catalyze taxa-4(20),11(12)-diene-5α-ol into the important precursor 2-debenzoyltaxane (Fig. 1). In total, 39 BAHD ACTs were found in the *T. cuspidata* transcriptome. In general, BAHD ACTs share 2 conserved regions, HXXXD and DFGWG motifs [12]. MEME analyses showed that among the 39 protein sequences, 8 sequences lacked an intact HXXXD motif or a DFGWG motif. The thirty-one BAHD ACTs with intact BAHD domains and some characterized BAHD ACTs from other plants were subjected to phylogenetic analyses, and all enzymes were divided into five distinct clades (Fig. 6b).

All BAHD ACTs from *T. cuspidata* were clustered into three clades, Clades I, IV and V. Clade V plays an important role in the acetylation of amino groups to form amides and transfer hydroxycinnamoyl- or benzoyl-CoAs. To date, all characterized BAHD ACTs involved in Taxol biosynthesis belong to Clade V. We found that 21 of 31 BAHD ACTs from *T. cuspidata* belonged to Clade V. Among them, TcuACT31 showed high similarity to the identified TAT (98%), which catalyzes taxa-4(20),11(12)-diene-5α-ol into taxa-4(20),11(12)-diene-5α-yl acetate in the Taxol pathway. However, we did not find the other four characterized BAHD ACTs in the *T. cuspidata* transcriptome. According to the real-time results, the four enzymes were expressed at substantially lower levels in *T. cuspidata* than TcuACT31 (Fig. 4c), which may lead to difficulty in their identification by sequencing analysis. The expression levels of twelve enzymes that were phylogenetically closest to the five characterized BAHD ACTs were analyzed using real-time PCR technology. Four members (*TcuACT8*, *13*, *17*, and *21*) had the same expression patterns as *TAT* and *TBT*, as their expression quantities in root tissue were similar to that in leaf tissue but significantly higher than that in stem tissue (Fig. 4c). In addition, three members *(TcuACT9*, *10*, 30) exhibited expression trends similar to those of *BAPT*, *DBAT* and *DBTNBT*, as their levels in roots were significantly higher than those in stems and leaves. We inferred that these seven BAHD ACTs are possible candidates for the Taxol pathway.

### Putative transcription factors regulating Taxol biosynthesis

**Fig. 7:**
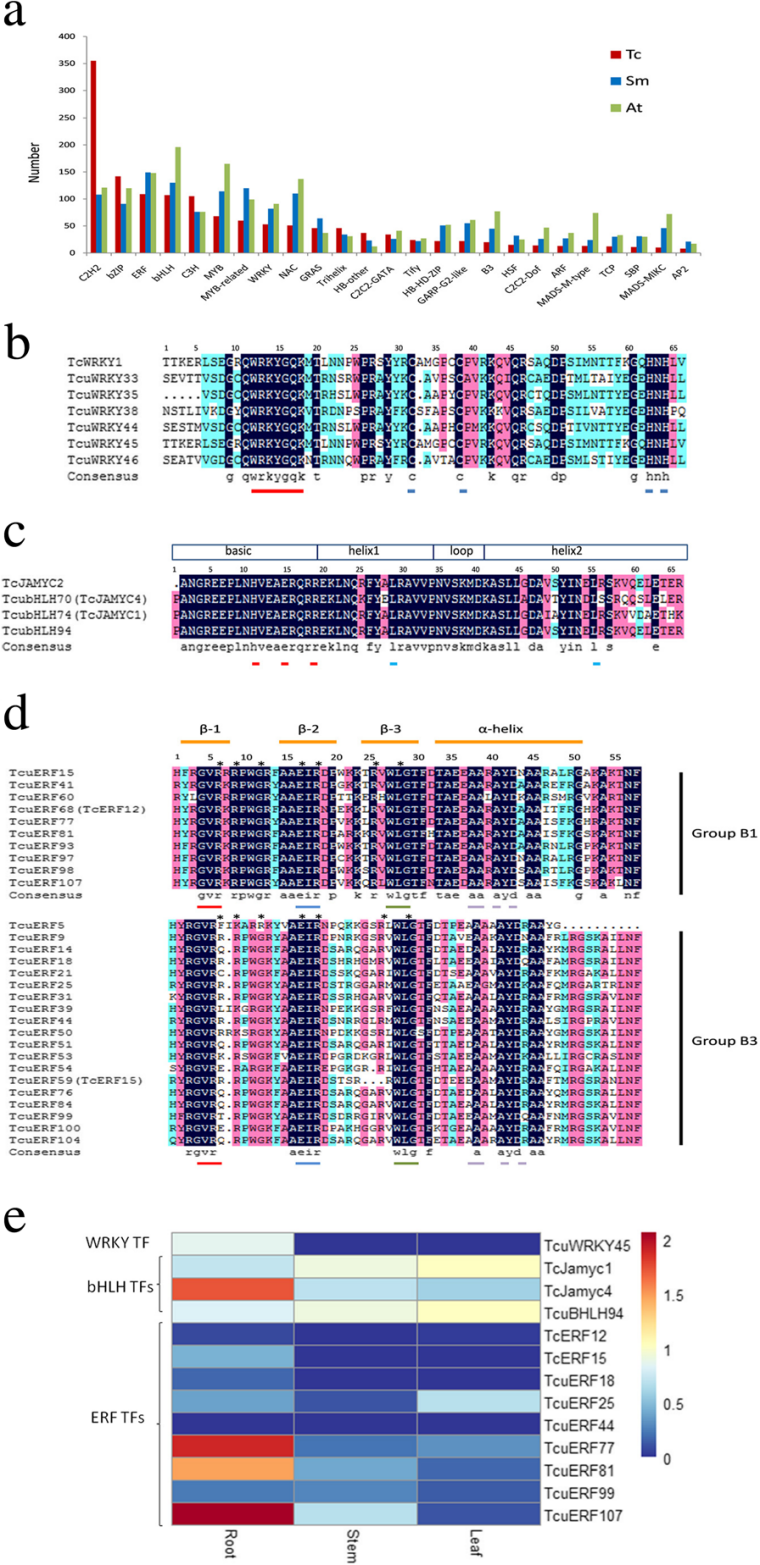
Transcription factor analysis. **a** Comparison of transcription factors in *T. cuspidata*, *A. thaliana*, and *S. miltiorrhiza*. **b**. Multiple sequence alignments of the WRKY domains of Group IIa WRKY proteins in *T. cuspidata*. Conserved WRKY amino acid signatures are indicated by short red lines, and zinc finger motifs are indicated by short blue lines. **c** Multiple sequence alignments of bHLH domains from subgroup IIIe bHLH proteins in *T. cuspidata*. The locations of the basic, helix, and loop regions within the bHLH domain are indicated in the scheme. The conserved amino acid signature of the basic motifs and the helix–loop–helix motifs are indicated by short red and blue short lines, respectively. **d** Multiple sequence alignments of AP2 domains from Group B1 and B3 ERF proteins in *T. cuspidata*. The three β-sheet regions and the α-helix region are labeled. The conserved amino acid signatures of β-1, β-2, β-3 and α-helix are indicated by short red, blue, green and purple lines, respectively. The essential residues for GCC-box binding in TcERF12 and TcERF15 are indicated by stars (*). **e** Expression analysis of identified TFs and candidate unigenes in the Taxol pathway by real-time PCR

We herein identified 1940 unigenes representing putative TFs distributed across 61 families and including bZIPs, bHLHs, WRKYs, and MYBs. The number of TFs is comparable to that of another diterpenoid-producing plant, *S. miltiorrhiza* (1948 TFs), and to that of the model plant *A. thaliana* (2357 TFs). As shown in Fig. 7a, we found 355 putative C2H2-type zinc finger-containing proteins in *T. cuspidata*, an obviously higher number than those in the angiosperm *A. thaliana* and *S. miltiorrhiza.* Plant C2H2 zinc finger proteins are mainly involved in the growth and development of plants at various stages and the regulation of gene expression under environmental stress, including extreme temperatures, salinity, drought, oxidative stress and excessive light [52]. The phenomenon of C2H2-type zinc finger protein families being more abundant than other families might be the result of more gene duplication events in the zinc finger-containing protein genes in *T. cuspidata* genomes during evolution, resulting in the rapid expansion of these genes, and they may have special functions in *T. cuspidata*. Seven TFs have been identified to be involved in Taxol pathway regulation, including one WRKY, three bHLHs, two ERFs and one AP2 TF [16–19].

WRKY proteins constitute one of the largest classes of TFs in plants and contain at least one highly conserved WKRY domain, which is essential for binding to DNA cis-elements [53]. More than ten plant WRKY TFs have been shown to regulate secondary metabolism, including TcWRKY1, PqERKY1, and CrWRKY1 [16, 54, 55]. TcWRKY1 from *T. chinensis* is the sole WRKY TF that regulates Taxol biosynthesis. TcWRKY1 was reportedly able to specifically bind to W-box elements (TTGAC(C/T)) within the *DBAT* promoter and activate *DBAT* expression [16]. A total of 36 proteins with complete WRKY domains were categorized into 3 groups and 7 subgroups (Additional file 10: Figure S7) according to Eulgem’s method [61]. We did not find the TF corresponding to TcWRKY1 in the *T. cuspidata* transcriptome, although six TcuWRKYs were divided into the same subgroup, Group IIa, with TcWRKY1 (Fig. 7b). Among them, TcuWRKY45 shared 88% identity with TcWRKY1 at the protein level and was expressed significantly more in roots than in stems and leaves (Fig. 7e). In addition, TcuWRKY45 had the same WRKY domain as TcWRKY1 (Fig. 7b), suggesting that it may also bind to the *DBAT* promoter, although its actual roles in Taxol biosynthesis require further elucidation.

bHLH proteins are the second largest class of TFs found in plants, and they all contain a bHLH DNA binding domain [57]. Three methyl jasmonate (MJ)-inducible bHLH TFs (TcJamyc1, TcJamyc2 and TcJamyc4) have been identified in *T. cuspidata* and shown to have a negative effect on Taxol biosynthesis [17]. Sixty-four TcubHLH TFs with intact bHLH domains were grouped into 13 subgroups by phylogenetic analysis according to Heim’s method [62]. (Additional file 11: Figure S8). TcJamyc1, TcJamyc2 and TcJamyc4 all belonged to subgroup IIIe, and three bHLH proteins from this transcriptome were divided into the same subgroup, among which TcubHLH74 and TcubHLH70 corresponded to TcJamyc1 and TcJamyc4, respectively. We did not find TcJamyc2 in this transcriptome dataset. TcubHLH94 showed the highest similarity to TcJamyc2 and had the same DNA binding domain, suggesting its possible role in regulating the Taxol biosynthetic pathway (Fig. 7c, and Additional file 11: Figure S8). Interestingly, as shown in Fig. 7e, TcJamyc4 showed its highest expression in roots, while TcJamyc1 and TcubHLH94 showed their lowest expression in roots. Therefore, the true roles of these TFs in Taxol biosynthesis need further study.

The AP2/ERF proteins are a large class of plant-specific TFs that share well-conserved AP2/ERF-type DNA binding domains [58]. To date, two ERFs (TcERF12 and TcERF15) from *T. chinensis* and one AP2 from *T. cuspidata* have been identified to regulate Taxol biosynthesis [18, 19]. A total of 119 AP2/ERF superfamily members in *T. cuspidata* were found in this transcriptome dataset, including 8 AP2s, 49 DREBs, 55 ERFs and 6 RAVs [63, 64]. We did not find the identified AP2, which belonged to group II with one AP2 domain, while all 8 of the AP2 family members in this study belonged to group I with 2 AP2 domains. Based on phylogenetic analysis, forty-three TcuERFs with complete domains were categorized into 6 subgroups (Additional file 12: Figure S9). Two identified TcuERFs, TcERF12 and TcERF15, belong to subgroups B1 and B3, respectively. Ten subgroup B1 members and nineteen subgroup B3 members were found in the *T. cuspidata* transcriptome. Among those TcuERFs, TcuERF34 and TcuERF31 are the enzymes corresponding to TcERF12 and TcERF15 in *T. cuspidata*, respectively (Fig. 7d). In Group B1, TcuERF77, 81 and 107 were closer to TcERF12; in Group B3, TcuERF18, 25, 44 and 99 were closer to TcERF15 (Additional file 12: Figure S9). Among those TcuERFs, TcuERF18, 44, 77, 81, and 107 had a similar expression pattern to TcERF12 and TcERF15, with the highest expression in the roots (Fig. 7e). These five ERFs are regarded as lead candidates for regulating Taxol biosynthesis in *T. cuspidata*. Further work is needed to verify the predicted roles of these ERFs in the regulation of Taxol biosynthesis.

## Discussion

Natural products are important sources for drug discovery, and more than one-third of all clinical drugs, including artemisinin, Taxol and vinblastine, are currently derived from natural products and their derivatives [59, 60]. Among these drugs, Taxol is the top-selling natural antitumor drug used for the treatment of several forms of breast, lung, liver, blood and gynecological cancers [4, 5]. However, tree cutting and resource destruction have led to serious shortages in Taxol resources [7]. Novel strategies to increase the Taxol supply and avoid disturbing natural resources are urgently needed, and elucidating the Taxol biosynthetic pathway will lay important groundwork for accomplishing this goal.

Due to its complex molecular architecture, featuring 11 chiral centers, the biosynthetic pathway of the tetracyclic diterpene compound Taxol is fairly complex [3]. The complexity of the structure results in diverse reactions being dedicated to hydroxylation, oxidation, epoxidation, acetylation, benzoylation and addition [11]. Starting from the metabolic branching point, cyclization of GGPP into taxa-4(20),11(12)-diene, at least 24 enzymes are known to be involved in Taxol biosynthesis (Fig. 1) [13, 65]. Due to its important role in clinical cancer treatment, Taxol biosynthesis has attracted the attention of scientists worldwide [7]. However, some pieces of the pathway remain unelucidated due to the extreme complexity of the Taxol biosynthesis process. According to the chemical reaction types, the missing enzymes are thought to mainly derive from the CYP450 and BAHD ACT families. For CYP450s, in the formation of 2-debenzoyltaxane, the CYP450 genes responsible for C1 hydroxylation, oxetane formation and C9 oxidation of the taxane core as well as the genes responsible for C2′ sidechain hydroxylation in the penultimate step remain unidentified. For ACTs, several acetylases necessary for the formation of the significant precursor 2-debenzoyltaxane have not been identified.

In this study, we developed a strategy to discover candidate genes involved in Taxol biosynthesis based on long-read transcriptome sequencing. First, we obtained nonredundant transcripts from PacBio Iso-Seq data after correcting with NGS reads and removing redundant transcripts. Then, Cogent software was used to generate unigenes by reconstructing UniTransModels. However, the reconstruction introduced some mistakes, such as obvious misclustering of transcripts from different genes, difficulty in distinguishing degraded transcripts and actual AS isoforms and deficiency in completely removing redundant reads, and these mistakes exert substantial negative effects on the downstream gene screen and discovery. To overcome the limitations of Cogent, we developed an in-house pipeline to correct and improve the Cogent output. In this pipeline, nonredundant transcripts were annotated, and associated transcripts (e.g., CYP450s or BAHD ACTs) were then subjected to further analysis. An alignment-based method was used to cluster and remove redundant reads to generate unigenes. For example, with this pipeline, 813 nonredundant transcripts annotated as CYP450 were clustered into 139 CYP450 unigenes, while with Cogent, among all CYP450 nonredundant transcripts, only 595 transcripts were constructed into the 136 UniTransModels, and 218 CYP450 transcripts were left. According to the manually curated CYP450 unigenes, two types of mistakes were evident in the Cogent output. One is that transcripts from one CYP450 were constructed into different UniTransModels, and the other is that transcripts from two CYP450s were constructed into one UniTransModel. Our in-house pipeline was more accurate than Cogent, partly because manual evaluation was used, and was thus utilized for the subsequent gene family analysis. The candidate genes related to Taxol biosynthesis were screened from these gene families by analyzing phylogenetic relationships, conserved motifs and expression profiles. This strategy avoids the mistakes introduced by short-read assembly in NGS and can produce assembly-free, highly accurate full-length and near full-length unigenes, which not only substantially contribute to specific gene cloning and characterization by molecular biology methods but also significantly improve the accuracy of gene annotation and gene expression quantification. With this strategy, we successfully identified 9 CYP450s and 7 BAHD ACTs as lead candidates for Taxol biosynthesis.

Long-read transcriptome sequencing also provided a substantial amount of genetic information for transcriptional and posttranscriptional regulation analyses, such as TFs, AS and lncRNAs. TFs that regulate transcriptional initiation by binding to cis-regulatory elements in promoters or enhancers are key players in regulating the biosynthesis of secondary metabolites [14]. In general, TFs can regulate multiple genes in a biosynthetic pathway simultaneously, making them attractive tools for improving the production of secondary metabolites [14, 15]. In total, we found 1940 unigenes encoding TFs in the *T. cuspidata* transcriptome, which is comparable to the number of TFs in another diterpenoid-producing plant, *S. miltiorrhiza*, and the number of TFs in the model plant *A. thaliana*. However, it is interesting that significant expansion of the C2H2 family was observed in *T. cuspidata*, suggesting that C2H2 TFs may play important roles in the survival of *T. cuspidata*. Three categories of TFs are reportedly involved in the Taxol pathway, including WRKY, bHLH and ERF/AP2 TFs. Using a strategy similar to that used for the discovery of enzymes involved in Taxol biosynthesis, 14 TFs were identified as candidates for the regulation of Taxol biosynthesis, including six WRKYs, one bHLH and seven ERFs.

AS is one of the most important posttranscriptional regulations and can affect gene expression by multiple regulatory mechanisms, and we herein observed that TFs easily underwent AS events in *T. cuspidata* [23]. In total, 60 AS events occurred in TFs, including WRKYs, bHLHs and bZIPs. In addition, we experimentally verified that the expression of several transcript isoforms exhibited a tissue-preferential pattern. Except for the isoform containing the intact ORF, all other isoforms have PTCs, suggesting that they are ultimately degraded via the NMD mechanism. The functional importance of many AS isoforms of TF genes has been characterized in other plants [66, 67]. Recent studies have shown that the AS of some TF genes generates small interfering peptides (siPEPs) that negatively regulate target TFs via peptide interference (PEPi), constituting self-regulatory circuits in the plant cold stress response [68]. The roles of AS in the regulation of Taxol biosynthesis should be further studied.

LncRNAs are another important group of regulators that play vital roles in plant stress responses [46, 69]. LncRNAs function mainly through transcriptional regulation and posttranscriptional regulation; in the former case, they interact with miRNA networks to regulate gene expression, and in the latter case, they interact with enhancers, promoters, and chromatin-modifying complexes to regulate gene expression [47]. Several lncRNAs have been functionally characterized in plant stress-responsive pathways [46]. For example, in wheat, Xin et al. [70] characterized TalnRNA27 and TalnRNA5, which are miRNA precursors, were upregulated under heat stress. Meanwhile, there is considerable evidence that many secondary products are capable of effectively responding to stress situations, such as heat, drought, salinity, and low temperature [71, 72]. Because lncRNAs have emerged as key regulatory molecules in plant stress responses, we speculated that lncRNAs may be involved in the regulation of secondary metabolism, but the question of whether lncRNAs can regulate Taxol biosynthesis needs further study.

In summary, compared to NGS, the TGS transcriptome provides substantially longer and more accurate sequence resources for gene discovery and AS analysis. However, for the Iso-Seq analysis of species without a reference genome assembly, the currently used software Cogent does not effectively cluster transcripts into unigenes or identify AS events. Here, we developed an in-house pipeline to analyze gene families and their AS events to screen candidate genes related to Taxol biosynthesis and regulation. This pipeline can plausibly be used for unigene generation and AS analysis at the gene family level, as it was successfully used to identify candidate genes related to the Taxol biosynthesis pathway. However, to analyze AS at the whole-transcriptome level, the effectiveness of Cogent needs to be improved or a more effective, novel software program needs to be developed to better analyze TGS transcriptome data without a reference genome.

## Conclusions

We developed an in-house pipeline to search for candidate genes involved in Taxol biosynthesis based on *T. cuspidata* transcriptome sequencing with PacBio SMRT technology. With this strategy, we identified 9 CYP450s and 7 BAHD ACTs as the lead candidate genes in the Taxol biosynthetic pathway and 6 TF genes that may regulate this pathway. We also investigated the coexpression of known genes in Taxol biosynthesis and elucidated the stem biosynthetic pathway based on the rule that genes in the same pathway are coexpressed. A coexpression analysis also suggested that the isoprene precursors for Taxol biosynthesis are mainly synthesized via the MEP pathway. In addition, we found and confirmed the existence of tissue-specific AS events, which represent a possible posttranscriptional mechanism in the regulation of Taxol biosynthesis. Our study provides not only a valuable resource for investigating novel genes in Taxol biosynthesis but also a practical procedure for screening candidate genes involved in secondary metabolite biosynthesis and analyzing AS events in organisms without reference genomes based on long-read transcriptome sequencing.

## Additional files

Additional file 1: **Table S1.** Proteins derived from GenBank used for the phylogenetic analysis. (XLSX 13 kb)

Additional file 2: **Table S2.** Primers used in this study. (XLSX 20 kb)

Additional file 3: **Table S3.** RNA-Seq data of identified genes and candidates for Taxol biosynthesis in *T. cuspidata*. (XLSX 46 kb)

Additional file 4: **Figure S1.** Venn diagram of unigene numbers of Iso-Seq from the KEGG, Swiss-Prot, Pfam, NT, NR, and KOG databases for *T. cuspidata. (TIF 1151 kb)*

Additional file 5: **Figure S2.** Identification of lncRNAs. (TIF 394 kb)

Additional file 6: **Figure S3.** GO annotation of unigenes. (TIF 6225 kb)

Additional file 7: **Figure S4.** Unigene functional classification by KEGG. The abscissa indicates the number of genes annotated to the pathway, and the ordinate indicates the subcategories. The pathway is divided into five categories in this analysis, including Cellular Processes, Environmental Information Processing, Genetic Information Processing, Metabolism, and Organismal Systems. (TIF 1344 kb)

Additional file 8: **Figure S5.** Functional classification of unigenes exhibiting increased expression in roots by KEGG analysis. (TIF 294 kb)

Additional file 9: **Figure S6.** Phylogenetic tree of 139 CYP450 proteins in *T. cuspidata*. The image shows an NJ tree made using CLUSTAL Omega (http://www.ebi.ac.uk/Tools/msa/clustalo/). The tree was drawn with Figtree v1.4.4 and labeled in GIMP2.8.2. (PNG 664 kb)

Additional file 10: **Figure S7.** Phylogenetic tree of WRKY domains among *T. cuspidata* and *A. thaliana*. Filled green diamonds represent unigenes in *T. cuspidata*, and the filled red circle indicates the identified gene in *Taxus*. (TIF 2580 kb)

Additional file 11: **Figure S8.** Phylogenetic analyses of bHLH domains in *T. cuspidata* and *A. thaliana*. Filled green diamonds represent unigenes in *T. cuspidata*, and the filled red circle indicates the identified gene in *Taxus*. (TIF 1382 kb)

Additional file 12: **Figure S9.** Phylogenetic analyses of ERF domains in *T. cuspidata* and *A. thaliana.* Filled green diamonds represent unigenes in *T. cuspidata*, and the filled red circle indicates the identified gene in *Taxus*. (TIF 2809 kb)

## Abbreviations

ACT: Acyltransferase
AHCT: Anthocyanin O-hydroxycinnamoyltransferase
AS: Alternative splicing
BAHD ACTs: BAHD acyltransferases
BAPT: Baccatin III-13-O-phenylpropanoyl transferase
BEAT: Benzylalcohol O-acetyltransferase
CCS: Circular consistency sequences
CNCI: Coding-Non-Coding Index
Cogent: Coding Genome Reconstruction Tool
CPC: Coding Potential Calculator
CYP450: Cytochrome P450
DAB: 10-deacetylbaccatin III
DAT: Deacetylvindoline 4-O-acetyltransferase
DBAT: 10-deacetylbaccatin III-10-O-acetyltransferase
DBTNBT: 30-N-debenzoyl-20-deoxytaxol-N-benzoyl transferase
DEGs: Different expressed genes
DMAPP: Dimethylallyl pyrophosphate
FDA: Food and Drug Administration
GGPP: Geranylgeranyl diphosphate
GGPPS: Geranylgeranyl diphosphate synthase
GO: Gene Ontology
HCBT: Anthranilate N-hydroxy- cinnamoyl/benzoyltransferase
ICE: Isoform-level clustering
IPP: Isopentenyl diphosphate
KEGG: Kyoto Encyclopedia of Genes and Genomes
KOG: EuKaryotic Ortholog Groups
MEP: Methylerythritol phosphate
ML: Maximum likelihood
MVA: Mevalonic acid
NGS: Next-generation sequencing
NJ: Neighbor-Joining
NMD: Nonsense-mediated decay
Nr: NCBI Non-redundant Protein
NSCLC: Nonsmall cell lung cancer
Nt: NCBI Non-redundant Nucleotide
ORF: Open reading frame
PAM: Phenylalanine aminomutase
PEPi: Peptide interference
PTC: Premature termination codon
siPEPs: Small interfering peptides
SMRT: Single molecular real-time
T2αH: Taxoid 2α–hydroxylase
T7βH: Taxoid 7β–hydroxylase
TAT: Taxadienol 5α-O-acetyl transferase
TBT: Taxane-2α-O-benzoyltransferase
TFs: Transcription factors
TGS: Third-generation sequencing
UniProtKB: UniProt Swiss-Prot
UniTransModels: Unique transcript models
