# Supplementary figures and images for: Iso-Seq analysis of the Taxus cuspidata transcriptome reveals the complexity of Taxol biosynthesis
Source: BMC Plant Biol. 2019 May 21;19:210. doi: 10.1186/s12870-019-1809-8 (PMC6530051; doi:10.1186/s12870-019-1809-8)

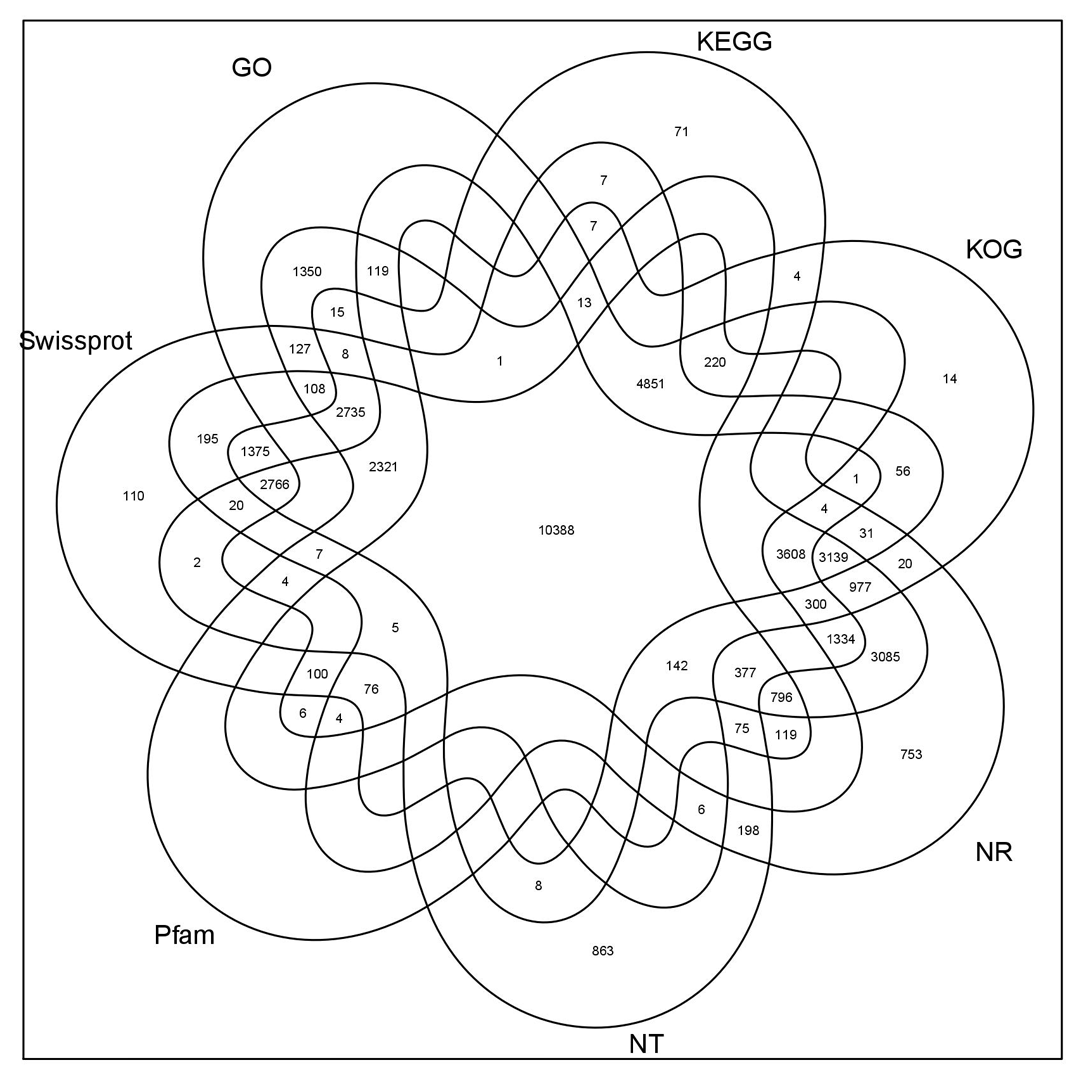

Supplement: Supplementary file 4 — Figure S1. Venn diagram of unigene numbers of Iso-Seq from the KEGG, Swiss-Prot, Pfam, NT, NR, and KOG databases for T. cuspidata. (TIF 1151 kb) [file 12870_2019_1809_MOESM4_ESM.tif]

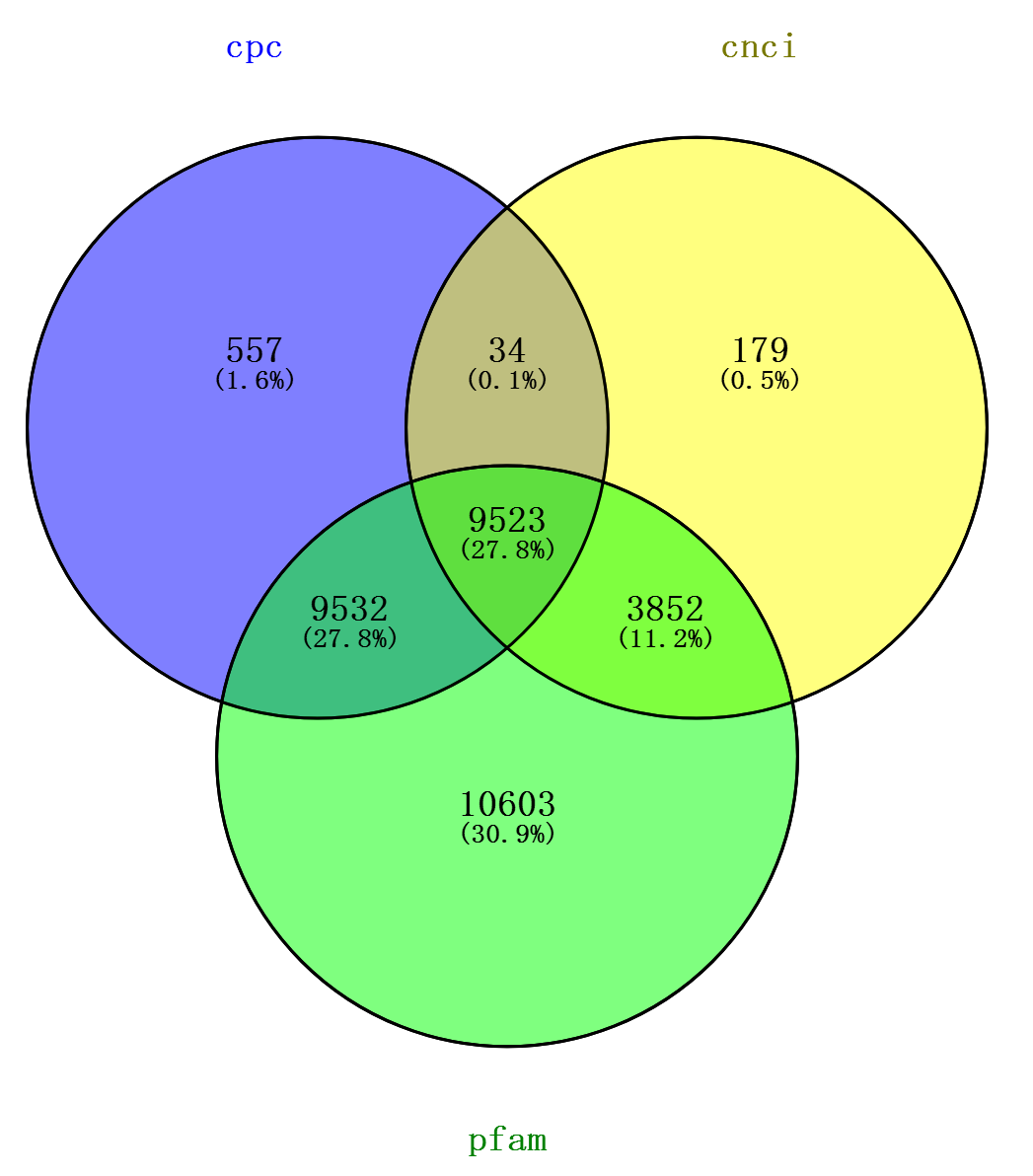

Supplement: Supplementary file 5 — Figure S2. Identification of lncRNAs. (TIF 394 kb) [file 12870_2019_1809_MOESM5_ESM.tif]

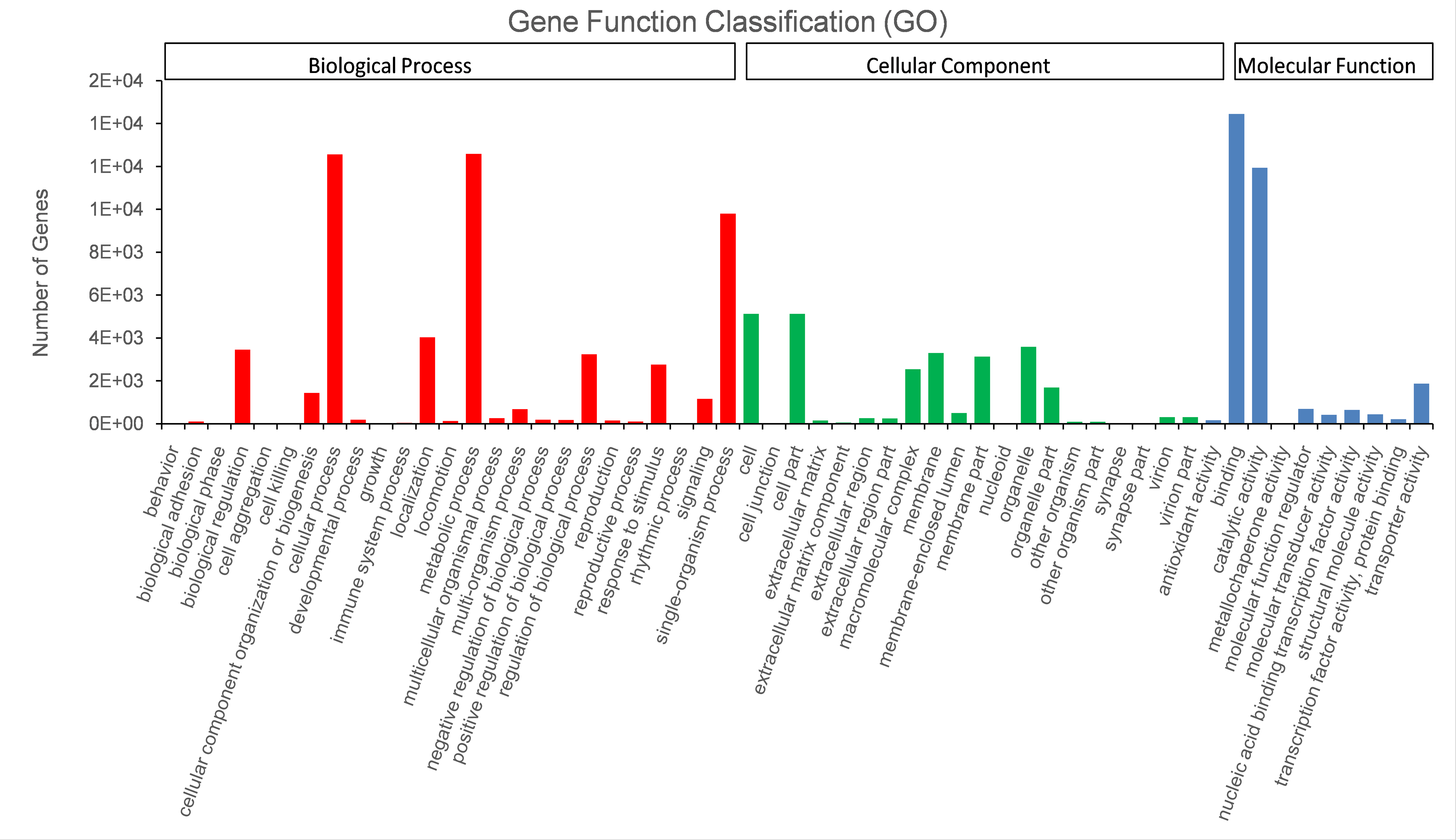

Supplement: Supplementary file 6 — Figure S3. GO annotation of unigenes. (TIF 6225 kb) [file 12870_2019_1809_MOESM6_ESM.tif]

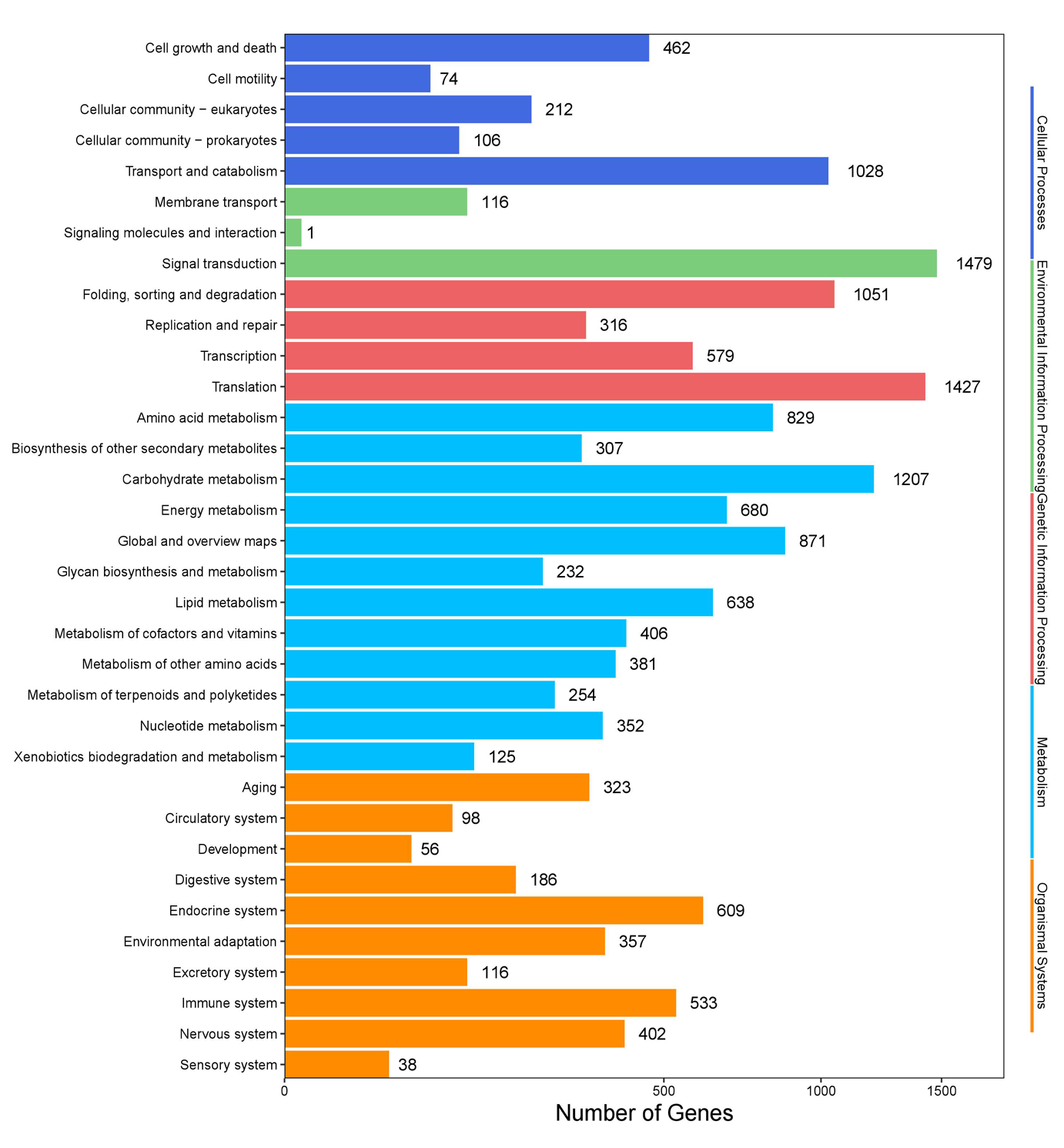

Supplement: Supplementary file 7 — Figure S4. Unigene functional classification by KEGG. The abscissa indicates the number of genes annotated to the pathway, and the ordinate indicates the subcategories. The pathway is divided into five categories in this analysis, including Cellular Processes, Environmental Information Processing, Genetic Information Processing, Metabolism, and Organismal Systems. (TIF 1344 kb) [file 12870_2019_1809_MOESM7_ESM.tif]

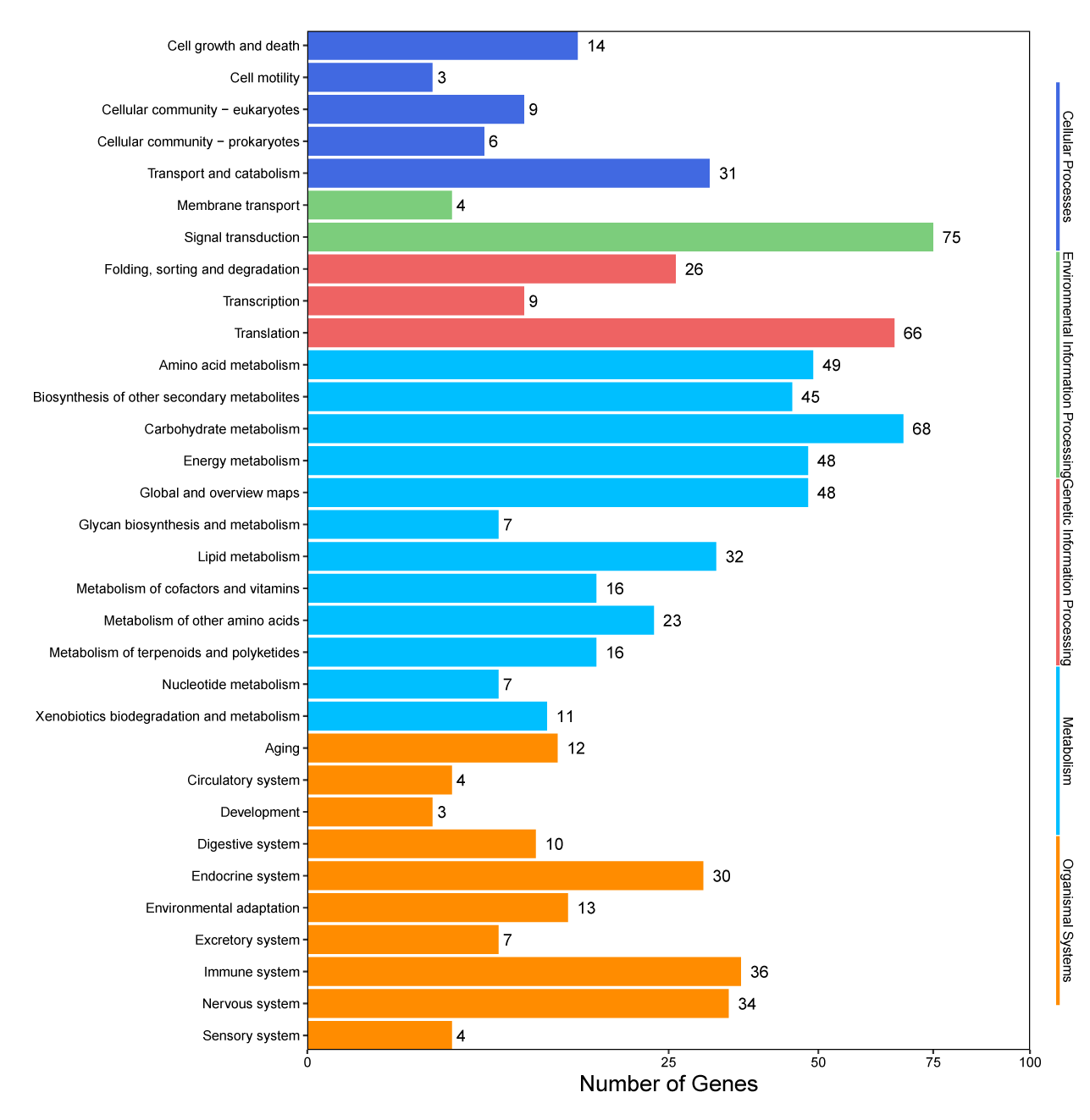

Supplement: Supplementary file 8 — Figure S5. Functional classification of unigenes exhibiting increased expression in roots by KEGG analysis. (TIF 294 kb) [file 12870_2019_1809_MOESM8_ESM.tif]

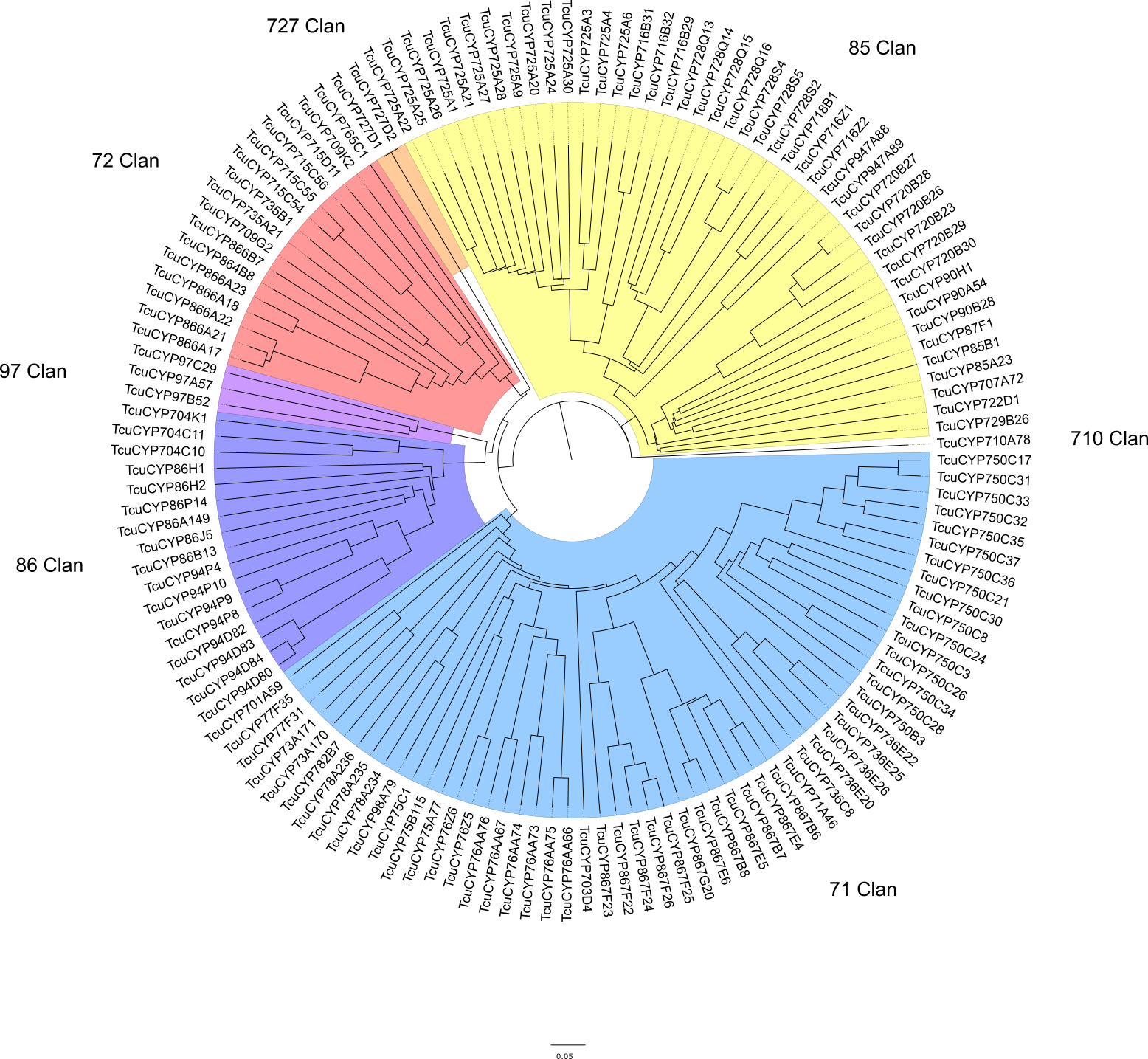

Supplement: Supplementary file 9 — Figure S6. Phylogenetic tree of 139 CYP450 proteins in T. cuspidata. The image shows an NJ tree made using CLUSTAL Omega (http://www.ebi.ac.uk/Tools/msa/clustalo/). The tree was drawn with Figtree v1.4.4 and labeled in GIMP2.8.2. (PNG 664 kb) [file 12870_2019_1809_MOESM9_ESM.png]

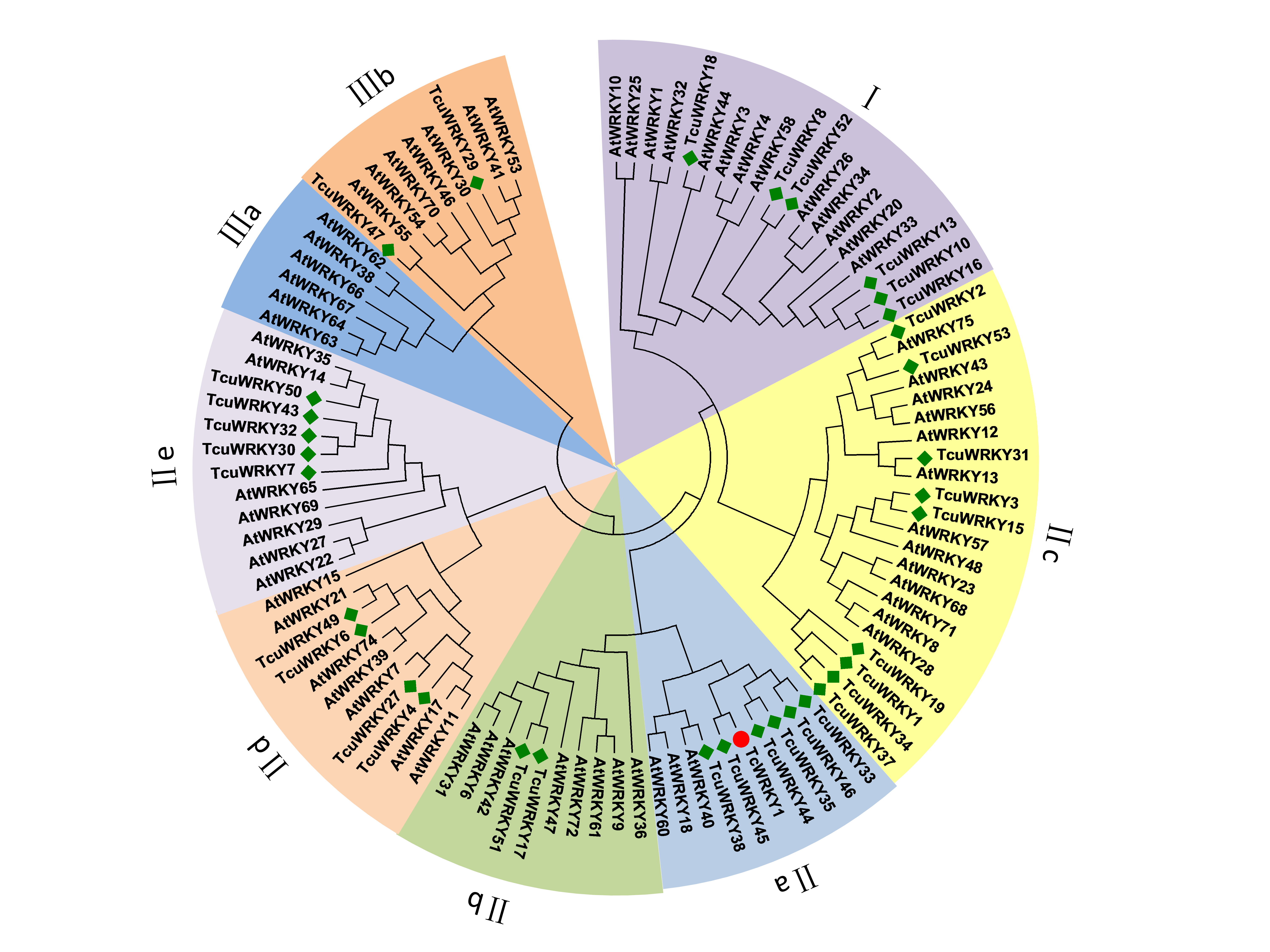

Supplement: Supplementary file 10 — Figure S7. Phylogenetic tree of WRKY domains among T. cuspidata and A. thaliana. Filled green diamonds represent unigenes in T. cuspidata, and the filled red circle indicates the identified gene in Taxus. (TIF 2580 kb) [file 12870_2019_1809_MOESM10_ESM.tif]

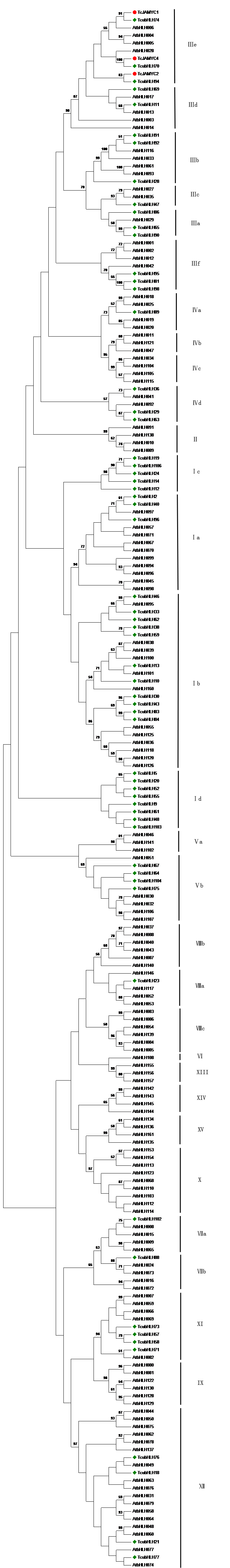

Supplement: Supplementary file 11 — Figure S8. Phylogenetic analyses of bHLH domains in T. cuspidata and A. thaliana. Filled green diamonds represent unigenes in T. cuspidata, and the filled red circle indicates the identified gene in Taxus. (TIF 1382 kb) [file 12870_2019_1809_MOESM11_ESM.tif]

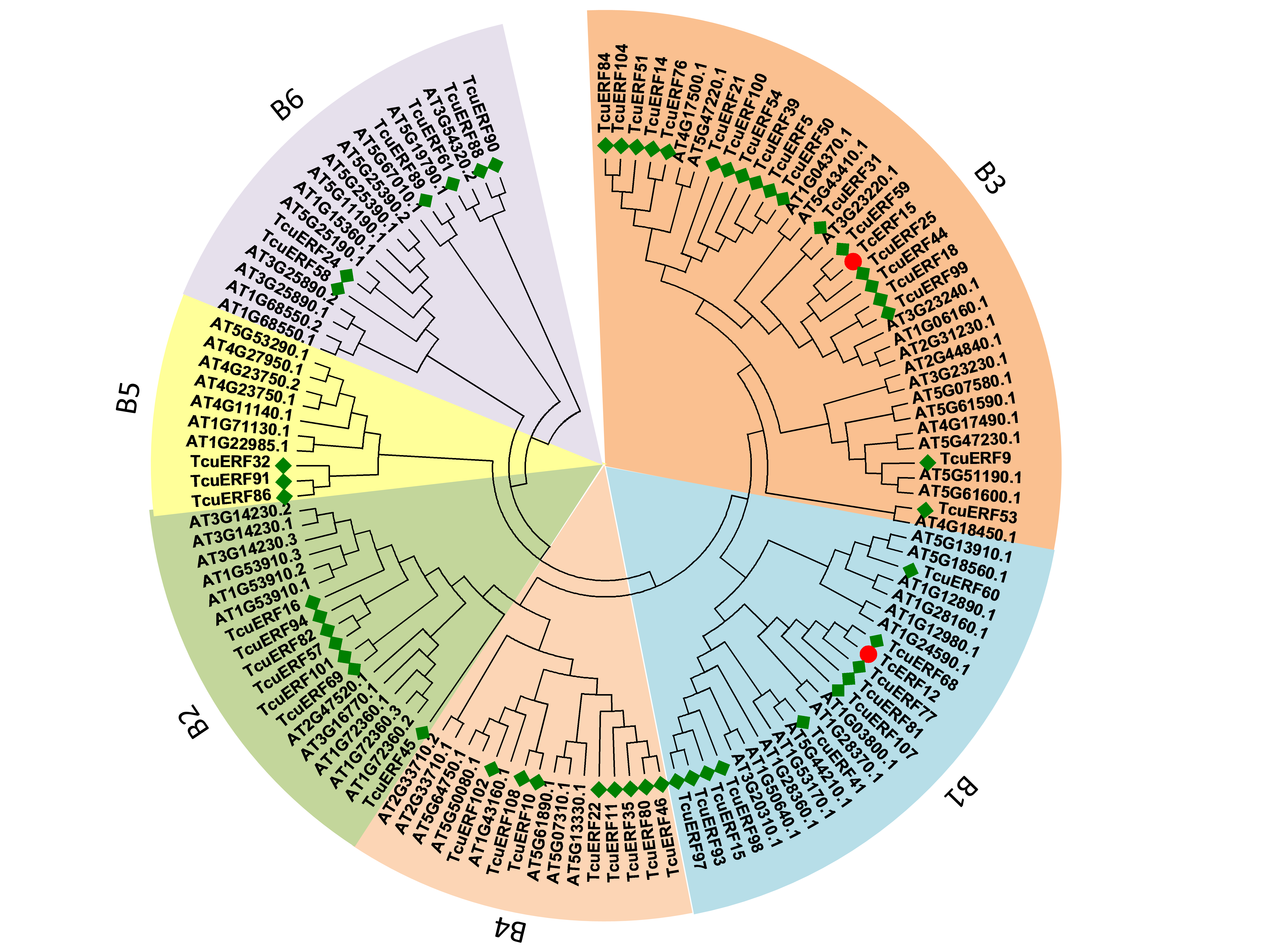

Supplement: Supplementary file 12 — Figure S9. Phylogenetic analyses of ERF domains in T. cuspidata and A. thaliana. Filled green diamonds represent unigenes in T. cuspidata, and the filled red circle indicates the identified gene in Taxus. (TIF 2809 kb) [file 12870_2019_1809_MOESM12_ESM.tif]
